# The Origin and Evolution of Baeyer—Villiger Monooxygenases (BVMOs): An Ancestral Family of Flavin Monooxygenases

**DOI:** 10.1371/journal.pone.0132689

**Published:** 2015-07-10

**Authors:** Maria Laura Mascotti, Walter Jesús Lapadula, Maximiliano Juri Ayub

**Affiliations:** IMIBIO-SL CONICET, Facultad de Química Bioquímica y Farmacia, Universidad Nacional de San Luis, San Luis, Argentina; Université Paris-Sud, FRANCE

## Abstract

The Baeyer—Villiger Monooxygenases (BVMOs) are enzymes belonging to the “Class B” of flavin monooxygenases and are capable of performing exquisite selective oxidations. These enzymes have been studied from a biotechnological perspective, but their physiological substrates and functional roles are widely unknown. Here, we investigated the origin, taxonomic distribution and evolutionary history of the BVMO genes. By using *in silico* approaches, 98 BVMO encoding genes were detected in the three domains of life: Archaea, Bacteria and Eukarya. We found evidence for the presence of these genes in Metazoa (*Hydra vulgaris*, *Oikopleura dioica* and *Adineta vaga*) and Haptophyta (*Emiliania huxleyi*) for the first time. Furthermore, a search for other “Class B” monooxygenases (flavoprotein monooxygenases –FMOs – and *N*-hydroxylating monooxygenases – NMOs) was conducted. These sequences were also found in the three domains of life. Phylogenetic analyses of all “Class B” monooxygenases revealed that NMOs and BVMOs are monophyletic, whereas FMOs form a paraphyletic group. Based on these results, we propose that BVMO genes were already present in the last universal common ancestor (LUCA) and their current taxonomic distribution is the result of differential duplication and loss of paralogous genes.

## Introduction

Baeyer—Villiger monooxygenases are a class of NADPH, flavin dependent enzymes, which catalyze the insertion of an oxygen atom between a C-C bond in ketones and aldehydes, as well as the oxidization of heteroatom-containing molecules (*i*.*e*: sulfides, amines and boron compounds) [1]. Extensive research has shown that these enzymes carry out the oxidation of a variety of substrates with exquisite chemo-, regio- and enantioselectivity [2]. Regarding BVMO’s physiological function, it has been demonstrated that some members of this family are involved in the synthesis of natural products, in the activation of prodrugs and in biodegradation processes. Some illustrative examples are the BVMOs from *Aspergillus fumigatus* Af293 [3], *Streptomyces argillaceus* ATCC 12956 [4, 5], *Pseudomonas* spp [6, 7], *Mycobacterium tuberculosis* [8] and *Acinetobacter radioresistens* [9]. Thus, it seems clear that BVMOs are not involved in primary cellular processes, but rather in a wide number of secondary metabolism pathways, meaning non-essential cellular processes [1, 10].

BVMOs are part of the so-called “Class B” (EC 1.14.13) monooxygenases along with flavoprotein monooxygenases (FMOs), *N*-hydroxylating monooxygenases (NMOs) and the YUCCAs [11]. All these enzymes share biochemical, mechanistic and sequence features. However, they present differences regarding substrate specificity and catalyzed reactions. FMOs have been described in all kingdoms of life. These enzymes use the electrophilic flavin C4a-hydroperoxide to oxygenate a wide range of carbon-bound nucleophilic nitrogens, sulfurs, and halides [12]. NMOs, which are more specific regarding the structure of substrates than other flavin monooxygenases, have been only detected in bacteria and fungi [13, 14]. Recently, the YUCCAs have been included as a subgroup of “Class B” monooxygenases. These are typically plant enzymes involved in the biosynthesis and metabolism of hormones and growth factors [15]. It is currently accepted that BVMOs occurrence is restricted to prokaryotic and some unicellular or modular eukaryotic organisms such as filamentous fungi [10]. Several bacterial BVMOs have been extensively studied from a catalytic perspective [16, 17]. Recently, a few fungal BVMOs have been also cloned and biochemically characterized. These are: the cycloalkanone monooxygenase (CAMO) from *Cylindrocarpon radicicola* [18], the Baeyer—Villiger monooxygenase Af1 (BVMO_Af1_) from *Aspergillus fumigatus* Af293[19] and four BVMOs from *Aspergillus flavus* NRRL3357 [20]. Furthermore, novel BVMOs from photosynthetic organisms, the rhodophyta *Cyanidioschizon merolae* and the bryophyta *Physcomitrella patents* have been characterized [21]. Some research dealing with the functional importance of conserved residues has been performed [22–24]. However, the origin and the evolutionary history of these enzymes have not been explored in deep.

The restricted and fitful occurrence of BVMO encoding genes throughout the tree of life could be explained by two different, but not mutually exclusive, evolutionary mechanisms; (i) the horizontal gene transfer (HGT) among evolutionary distant species [25–27] or (ii) the differential duplication and loss of paralogous genes in species derived from ancestral taxa [28–30]. For non-essential genes, genetic drift is a major force that molds their evolution leading to its fixation or loss from genomes [31, 32].

In this work we have analyzed the taxonomic distribution of BVMOs across the tree of life and studied their phylogeny in the context of the whole “Class B” monooxygenases. Based on this, we have proposed an evolutionary model explaining the emergence and current distribution of BVMO genes.

## Results

### Distribution of BVMO encoding genes throughout the tree of life

In this work 98 BVMOs were newly identified: 69 sequences belong to eukaryotic organisms, 26 to Bacteria and 3 to Archaea. These sequences were identified by an iterative homology searching process of three consecutive steps aimed to detect both, close and remote homologues (see Materials & Methods). Distribution of sequence identity among the identified BVMOs is presented in S1 Fig. All the sequences displayed the BVMO hallmarks: the two Rossmann motifs (GxGxx[G/A]) flanking the two BVMO fingerprints ([A/G]GxWxxxx[F/Y]P[G/M]xxxD and FxGxxxHxxxW[P/D] [22, 24]), confirming unambiguously the BVMO-nature of the selected sequences (S2 Fig). Recently Rebehmed *et al* described a refinement of the second BVMO fingerprint adding a Thr residue to the consensus (FxGxxxHTxxW[P/D]) [23]. We found that this residue was only partially conserved. This could be due to the fact that our dataset includes more divergent sequences.

**Fig 1:**
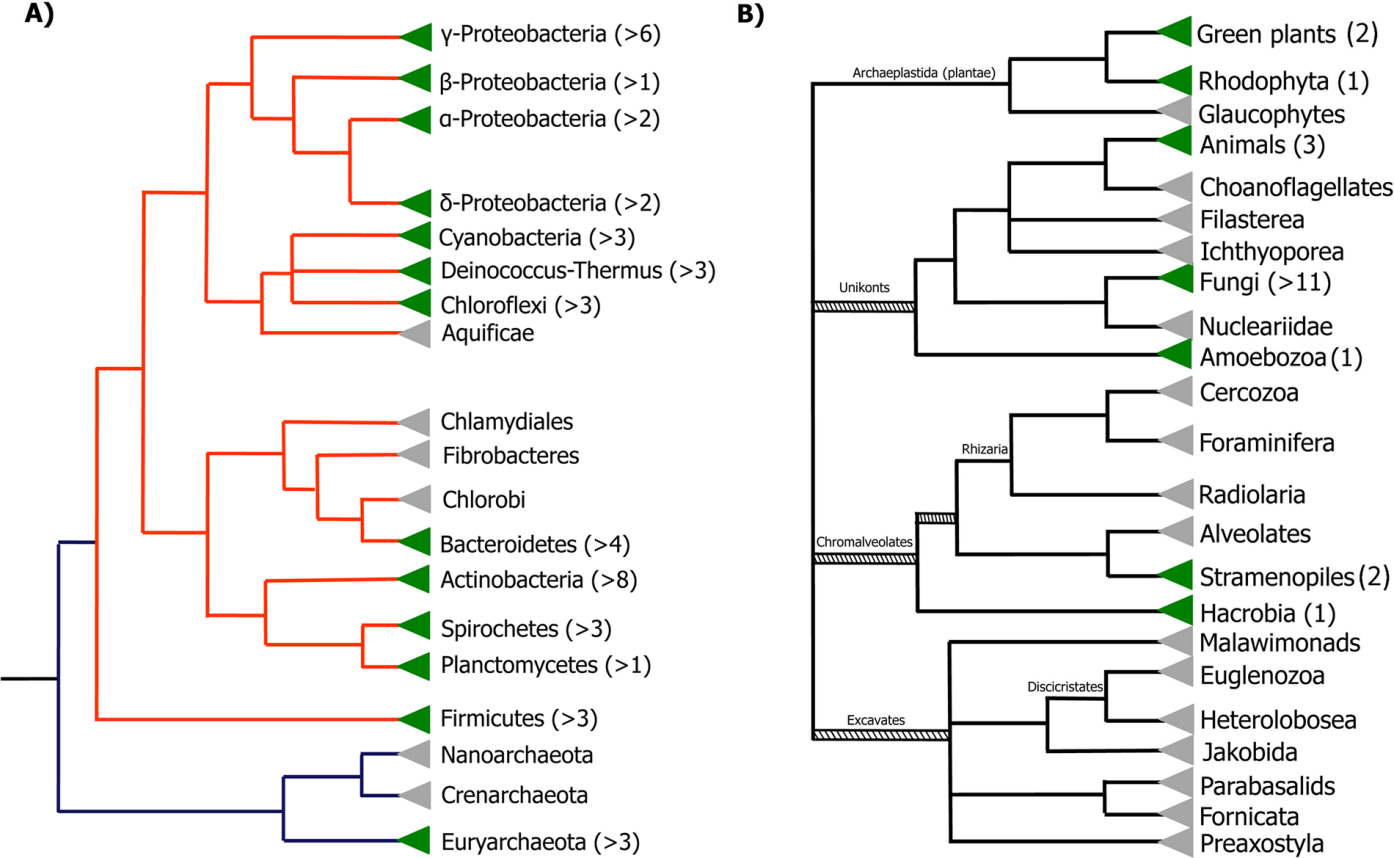
Taxonomic distribution of BVMO genes. **A.** Distribution of BVMO encoding genes in genomes from Bacteria and Archaea domains. **B.** Distribution of BVMO encoding genes in genomes from Eukarya domain. Green triangles show clades containing BVMOs; grey triangles show clades where BVMOs were not detected. Numbers in brackets show the number of different species where BVMOs were found. The tree topology is based on the tree of life web project (Maddison DR and Schulz KS (eds.) 2007. Available at: http://tolweb.org (Accesed 17 November 2014)).

Our research revealed a fitful taxonomic distribution, including the presence of BVMO encoding genes in unexpected taxa. All phyla of the domain Bacteria were evaluated for the presence of *BVMO*-encoding sequences. Already characterized BVMOs come from species belonging mainly to β and γ-Proteobacteria (*Pseudomonas* spp, *Acinetobacter* spp, *Comamonas* spp, among others) and Actinobacteria (e.g. *Rhodococcus* spp, *Mycobacterium* spp., *Gordonia* sp.). New BVMOs were found in almost all groups excepting Acidobacteria, Aquificae, Thermotogae, Fusobacteria, Chlamydiae, Chlorobi and Chloroflexi. Thus, only two or three sequences belonging to different species were selected as representatives of each phylum to be included in further phylogenetic analyses. In the domain Archaea, BVMOs were found only in the phylum Euryarchaeota, in three species belonging to the class Halobacteria, but were not found in the phyla Crenarchaeota or Korarchaeota (Fig 1A).

In the domain Eukarya, new BVMO sequences were detected in several fungal species belonging to phyla Basidiomycota and Ascomycota, as it was expected from previous reports [19, 20]. Interestingly, a high number of sequences were found in several fungal species such as *Aspergillus flavus* (25), *Aspergillus niger* (> 9) and *Aspergillus fumigatus* (8). Additionally, we found preliminary evidence *in silico* suggesting the presence of one BVMO gene in the monocot *Hordeum vulgare*. This sequence derived from a cDNA library and no matching sequences were found in the *H*. *vulgare* genomic database. Therefore, by performing PCR experiments, we determined that this sequence does not belong to *H*. *vulgare* genome (S3 Fig).

On the other hand, five *BVMO* encoding sequences were detected in the eukaryotic haptophyta *Emiliania huxleyi*. The presence of these sequences was checked by PCR. The paralogous sequences *Ehux1-2* and *Ehux3-4* were successfully amplified from *E*. *huxleyi* genomic DNA (S4 Fig). However, the sequence *Ehux5* could not be amplified, which could be related to the genomic intraspecific variation among *E*. *huxleyi* strains [33]. Likewise, BVMO sequences were identified in several Eumetazoa species: *Hydra vulgaris* (1) belonging to the phyla Cnidaria, and in the bilaterian animals *Oikopleura dioica* (2) and *Adineta vaga* (1) (Fig 1B). A surprising fact was the finding of four BVMO encoding genes in the draft genome of the Tibetan antelope *P*. *hodgsonii*. Therefore the genomic context of these genes was studied. The results strongly suggested that the BVMO-containing contigs came from bacterial contamination somewhere in the pipeline of the sequencing project [34] (see S1 File for details). Our observation is in agreement with the recent report of other bacterial contaminant sequences in the *P*. *hodgsonii* database [35].

In summary, we have demonstrated the presence of *BVMO* genes in Haptophyta and Metazoa. Moreover, during the revision process of this article, new BVMO sequences were detected in the stramenopiles *Saprolegnia diclina* (XP_008604020.1) and *S*. *parasitica* (KDO22379.1), the amoebozoa *Acytostelium subglobosum* (GAM26105.1) and the chlorophyta *Monoraphidium neglectum* (KIZ04091.1) (Fig 1B). Considering this new picture of BVMOs distribution, it can be stated that the presence of these enzymes is not restricted to a limited fraction of species as it was presumed before, displaying a wider dissemination along the three life domains.

### Phylogenetic analysis of BVMOs family

**Fig 2:**
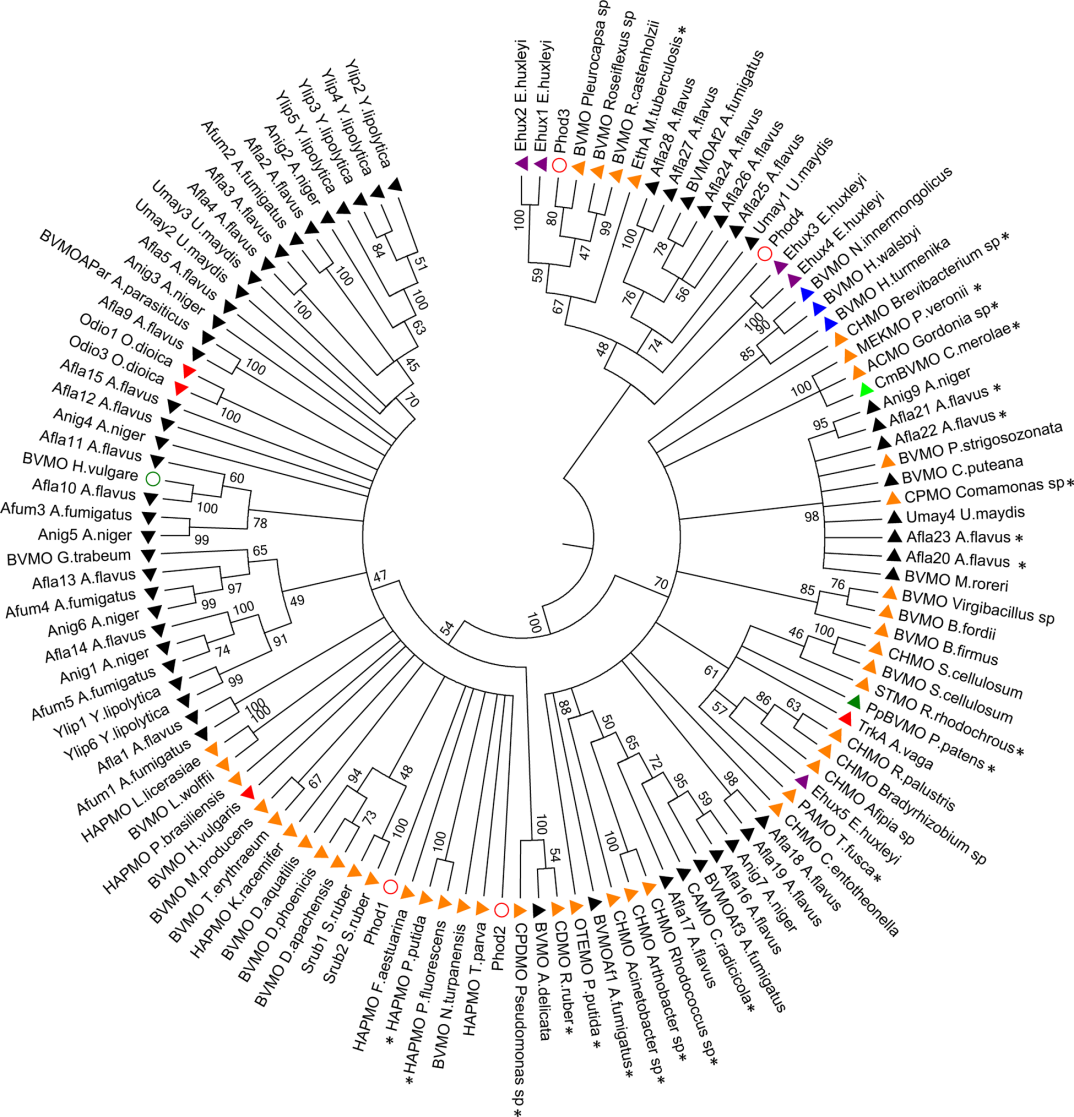
Phylogenetic analysis of BVMOs protein-family by Maximum Likelihood (ML) method. The tree was constructed by using the ML method, employing the alignment constructed with MAFFT 7 on-line tool and the best model parameters calculated with ProTest 3.4. Evolutionary analyses were conducted in PhyML 3.0 on-line server. Bootstrap values (> 45) are shown next to the branches. Colored triangles show the source of BVMO genes as follows: Fungi (black), Bacteria (orange), Green plants (dark green), Rhodophyta (light green), Metazoa (red), Haptophyta (purple) and Archaea (blue). Empty circles indicate BVMO encoding sequences where strong evidences of contamination artifacts were found: *Pantholops hodgsonii* (red) and *Hordeum vulgare* (dark green). Sequences marked with asterisks have been previously characterized.

To understand the origin and evolutionary relationships among *BVMO* genes from taxonomically distant organisms, a phylogenetic analysis was conducted employing the Maximum Likelihood (Fig 2) and Bayesian inference methods (S5 Fig). The obtained trees were equivalent regarding the distribution and the composition of clades. The branches were supported by high bootstrap (BS) and posterior probability (PP) values. Moreover, the addition or deletion of various sequences from the protein dataset did not affect the global topology.

A very complex topology was noticed, being observed that closely phylogenetically related genes belong to genomes from evolutionary largely distant organisms. For instance, a clade of BVMOs with good statistic support (BS: 61, PP: 0.9582) includes sequences from gram positive (*Thermobifida fusca*, *Rhodococcus rhodochrous* and *R*. *palustris*) and gram negative (*Afipia* sp., *Bradyrhizobium* sp. and *Sorangium cellulosum*) bacteria, a haptophyta (*Emiliania huxleyi*), a bryophyta (*Physcomitrella patens*) and a metazoan (*Adineta vaga*). This implies that an ancestral gene gave rise to BVMO genes belonging to largely divergent species. As it can be clearly seen in the phylogenetic trees, BVMO genes from bacteria and eukaryotes are polyphyletic (Fig 2 and S5 Fig). In addition, there are many examples of organisms harboring several BVMO genes (*e*.*g*. *Yarrowia lipolitica*), whereas genomes of closely related species (*e*.*g*. *Saccharomyces cerevisiae*) are devoid of BVMO sequences, indicating the occurrence of gene loss events.

When analyzing metazoan BVMOs, it appears that in the common ancestor with fungi, meaning the Opisthokonta, at least two BVMO paralog genes were present. This can be inferred because two different clades harbor metazoan/fungal pair sequences, such as: TrkA (*Adineta vaga*)/Afla18 (*A*. *fumigatus*) and Odio1 (*Oikopleura dioica*)/BVMO_Apar_ (*A*. *parasiticus*). Likewise, in the haptophyta *Emiliania huxleyi* five different paralogs are observed, of which two pairs may have raised by recent duplications, rendering the paralogous couples *Ehux1*-*Ehux2* and *Ehux3*-*Ehux4*. In consequence in the Hacrobia ancestor at least three different BVMO genes may have been already present. This line of evidence can be extended to propose that in the common ancestor between Bacteria, Archaea and Eukarya there were at least two paralogous BVMOs. Then, Bacteria and Eukarya conserved both of them. On the other hand in Archaea only one was retained, according to the current data. These observations are compatible with the occurrence of multiple gene loss and duplication events through different evolutionary lineages.

### Evolutionary origin of metazoan BVMOs

The discovery of novel BVMOs encoded in metazoan genomes is a striking result. According to our analyses we propose that the origin of these genes comes from an Opisthokonta ancestor. This is also supported by the fact that a close neighbor of animals is the fungi lineage, where BVMOs are widely disseminated. It has been claimed BVMO *TrkA* from *Adineta vaga* was acquired by horizontal gene transfer from bacterial genomes [36]. We addressed this possibility by performing homology searching and genomic context analyses (See S2 File for details). We found that although TrkA resembles bacterial sequences, it also displays high similarity to sequences from the metazoans *Oikopleura dioica* and *Hydra vulgaris*, whose genomes have been deposited in 2010 and 2013, respectively. The phylogenetic reconstruction (Fig 2) depicts that, even when TrkA sequence shows high identity to bacterial proteins it is part of a clade including other eukaryotic genes (from *E*. *huxleyi and P*. *patents*). Moreover, a similar situation is observed when analyzing the phylogenetic relationships of the other identified BVMO genes from metazoans, *e*.*g*.: *H*. *vulgaris* and *O*. *dioica*. Therefore, the presence of a BVMO gene in *A*. *vaga* is an infrequent, but not unique, case among metazoans. Altogether, these data suggest that the evolutionary history of this gene involves an ancestral origin along with differential duplication and loss events through evolution, strongly challenging the hypothesis of its acquisition by HGT.

### Phylogenetic relationships of BVMOs with other “Class B” flavin monooxygenases

**Fig 3:**
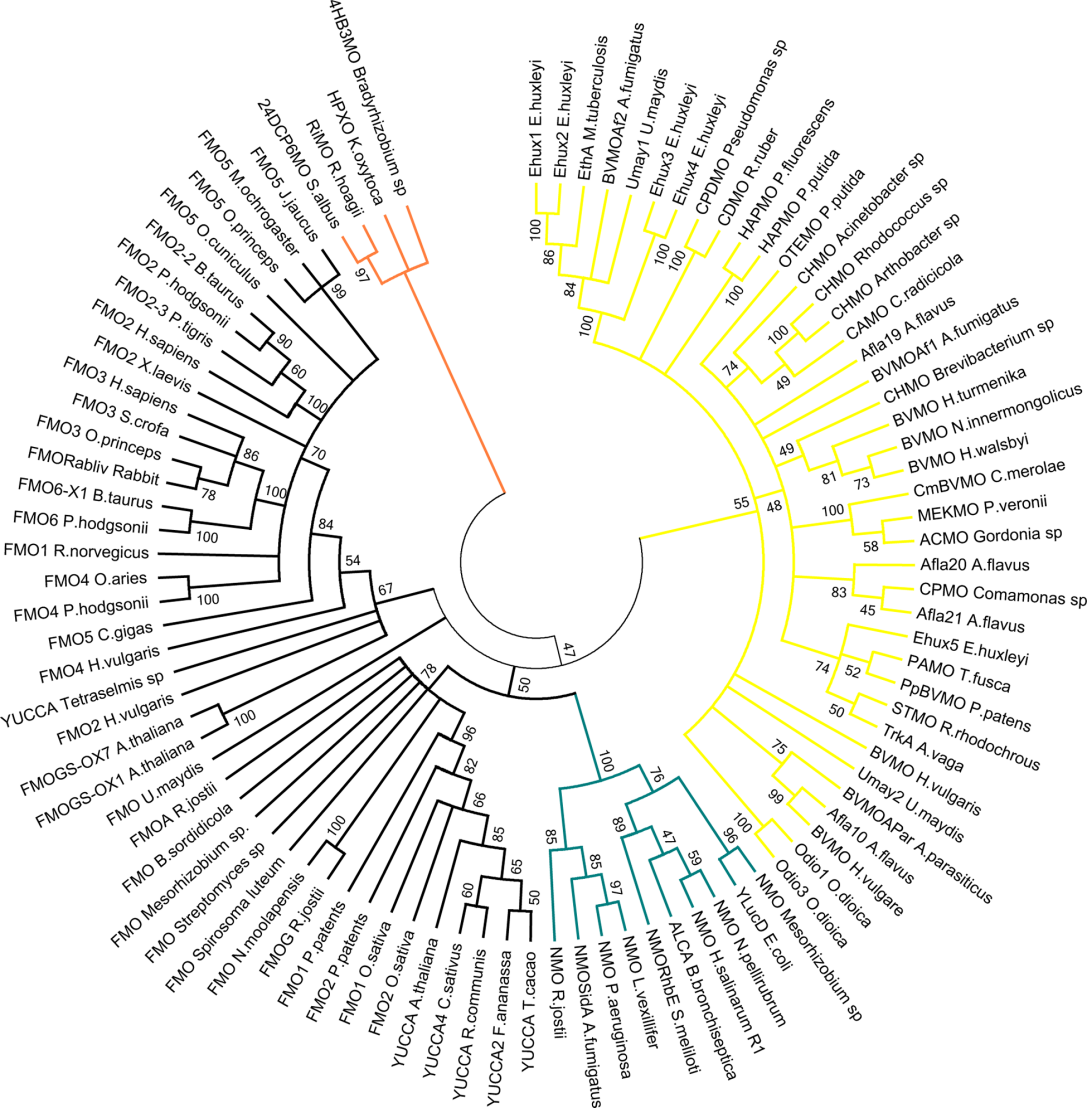
Rooted phylogenetic tree of flavin monooxygenases “Class B” by Maximum Likelihood (ML) method. The tree was constructed by using the ML method, employing the alignment constructed with MAFFT 7 on-line tool and the best model parameters calculated with ProTest 3.4. Evolutionary analyses were conducted in PhyML 3.0 on-line server. Bootstrap values (> 45) are shown next to the branches. Colored branches display: BVMOs (yellow), NMOs (blue) and FMOs (black). As outgroup, hydroxylases belonging to “Class A” flavin monooxygenases were employed (orange).

BVMOs, along with FMOs, NMOs and the recently included YUCCAs [11], belong to the “Class B” of flavin monooxygenases. These proteins share some basic biochemical features and the presence of two Rossmann motifs on their sequences. To elucidate their evolutionary history, a survey of novel sequences in Bacteria, Archaea and Eukarya was conducted by homology searching. Then, phylogenetic analyses including the retrieved sequences as well as enzymes previously characterized at the biochemical level for each family [37, 38] were performed. “Class A” monooxygenases were included as outgroup in order to root the tree (Fig 3 and S6 Fig). The topology of the tree proved to be highly robust, since by employing ML or Bayesian inference methods the clades were formed by the same sequences and the branches were supported by high BS and PP values.

**Fig 4:**
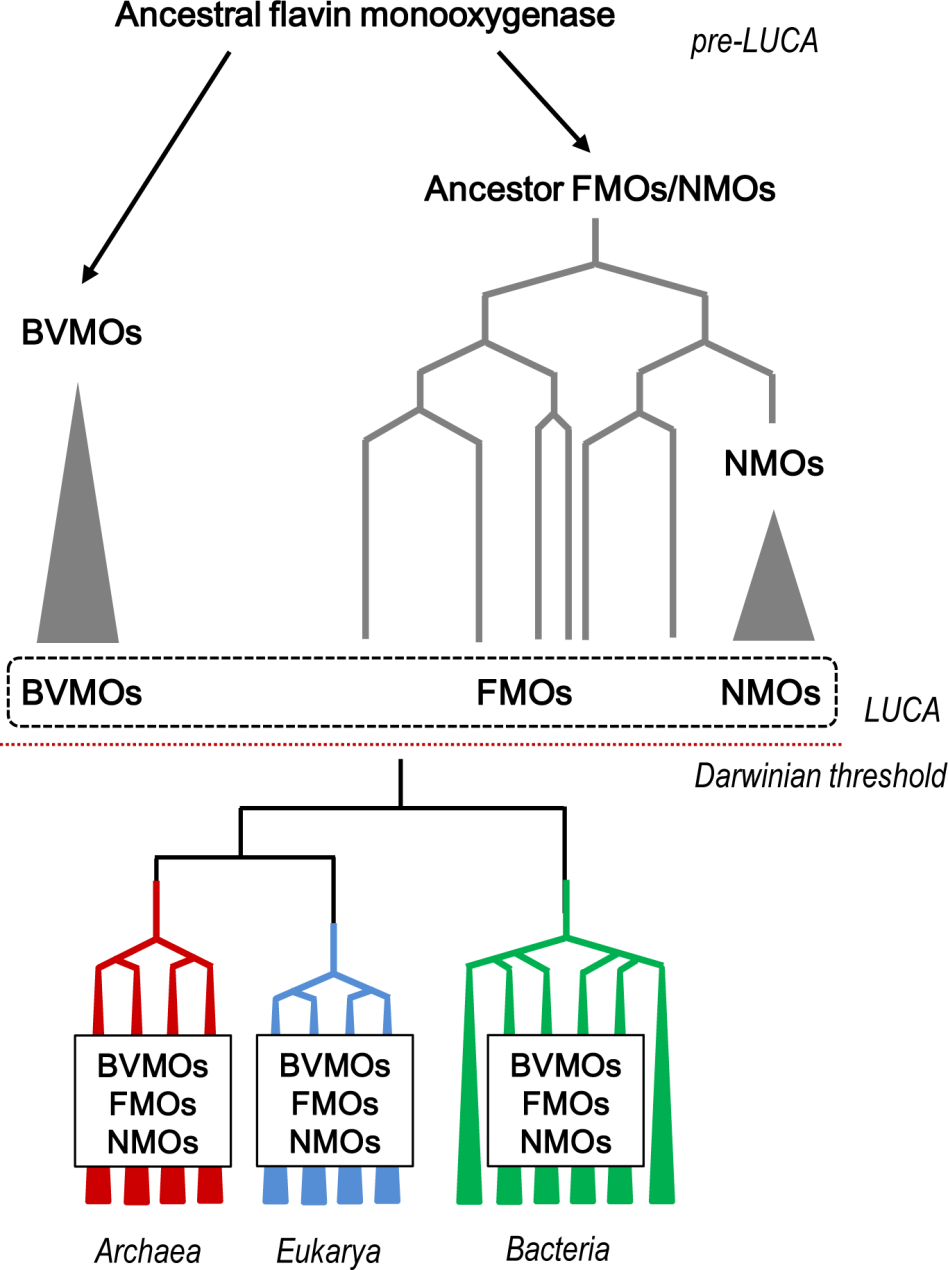
Scheme of the proposed model for the evolutionary history of “Class B” flavin monooxygenases. Hypothetical ancestral flavin monooxygenases genes were present in a pre-LUCA time. These genes gave origin to the monophyletic group of BVMOs and to a group of FMOs/NMOs-like genes, which later originated the monophyletic group of NMOs and a paraphyletic group of modern FMOs. We propose that BVMOs, FMOs and NMOs were already present in LUCA. After crossing the Darwinian threshold different paralogous of flavin monooxygenase genes were inherited by the three life domains: Bacteria, Archaea and Eukarya.

The topology of the trees indicated that BVMOs and NMOs are monophyletic groups of proteins, while FMOs and YUCCAs form a paraphyletic group. The rooting of the tree using “Class A” monooxygenases as outgroup, suggests that the origin of BVMOs predates NMOs (Fig 3 and S6 Fig). On the other hand, it was observed that YUCCAs are clustered with FMOs. Thus, although it has been recently proposed that YUCCAs and FMOs are different flavin monooxygenases groups [11], our results suggest that YUCCAs are part of FMOs group, in agreement with the report of Fraaije *et al* [22]. Additionally, NMOs were found in lineages other than Bacteria and Fungi [12], such as Metazoa and Archaea (S7 Fig). This is similar to the pattern observed for BVMOs and FMOs, suggesting that these three flavoproteins families (FMOs, NMOs and BVMOs) constitute ancient groups whose common ancestors must have been already present before the rising of the three life domains (Fig 4 and Discussion).

## Discussion

The evolution of enzyme families has been classically studied under the assumption that enzymes belonging to secondary metabolism have been originated in a late evolutionary stage “in order to cope with” specific problems of modern organisms [39, 40]. In the particular case of BVMOs, it has been proposed that its distribution is limited to Fungi and Bacteria and, even more, its evolution would be linked to its taxonomic distribution [20]. In contrast to this, we have demonstrated that these enzymes are widely distributed across the tree of life, finding novel sequences in Haptophyceae, Metazoa, Archaea, Stramenopiles and Amoebozoa. Therefore, it can be predicted that, as new genomic information of phylogenetically distant species becomes available, our knowledge about the distribution of BVMOs across extant organisms will be further modified.

On the basis of the evidences obtained in this work, we propose that BVMOs constitute a very ancient group of enzymes, whose evolution has been mainly shaped by genetic drift, yielding gain and loss of paralogs at different stages of the evolution of organisms. This hypothesis explains the unexpected associations among largely divergent species in the BVMOs tree. Besides, this argument takes into account that inside some lineages, BVMOs may have become useful resources (*e*.*g*: in defense metabolite synthesis) preventing gene loss events by genetic drift during the evolution of these species. Such could be the case of the genus *Aspergillus* or *Rhodoccocus*, where a significant number of BVMO encoding genes have been detected [3, 41–43].

The occurrence of HGT events requires several filters (the cellular and nuclear membranes, being incorporated to the genome, the germline barrier) to be passed. In addition, phylogenetic anomalies can be explained by HGT as well as by differential segregation of paralogous. Therefore, the real impact of HGT on the evolution of eukaryotic genomes is strongly debated [27, 44, 45]. Regarding to BVMO genes, although HGT cannot be completely excluded, the evidences found in our work suggest that the more parsimonious scenario explaining the occurrence of BVMOs in metazoa involves gene duplication and differential loss of paralogous genes. This is supported by: (i) the low sequence identity of metazoan sequences to BVMOs from other taxa thus being difficult to postulate the source organisms of HGT, (ii) the non-similar genomic contexts among species grouped in the same clade of BVMOs tree and (iii) the phylogenetic relationships among metazoan/non-metazoan BVMOs which are not consistent with HGT model.

Concerning the biochemical features, it has been largely proposed that BVMOs sharing biocatalytic properties cluster together [46, 47]. However, we have not found further evidence to support this idea. This is because only a few BVMOs possess known physiological substrates [3, 48, 49] (S3 File). Additionally, we have not found proofs suggesting that BVMOs may cluster together according to their domain architecture (S4 File).

Based on our results, we propose a model explaining the evolution of BVMOs in the context of a *last universal common ancestor* (LUCA) conceived as a genetically redundant complex community of organisms [50–52]. In a pre-LUCA time, there may have been ancestral flavin monooxygenase genes which gave origin to a monophyletic group of BVMOs and a paraphyletic group of FMOs. NMOs arose later from FMOs as a monophyletic group and, until LUCA time, FMOs remained as a paraphyletic group. After crossing the “Darwinian threshold” and the consequent crystallization of the three life domains, multiple paralogous genes of *FMOs*, *NMOs* and *BVMOs* were inherited by each domain (Fig 4). Although we do not know how refined these ancestral enzymes were, based on the biochemical features of modern FMOs, BVMOs and NMOs, it is tempting to hypothesize that FMOs are widespread among organisms due to their wider reaction scope as well as substrate acceptance [53]. Therefore, FMOs may display more universal functions such as detoxification of xenobiotic compounds, while NMOs and BVMOs would be more specific enzymes, since they are stricter regarding both its substrate and reaction scope [54].

## Conclusions

1. 98 newly-identified BVMOs of Bacteria, Archaea and Eukarya are described in this work.
2. BVMOs constitute an ancestral family of proteins, irregularly and widely spread among the tree of life.
3. BVMOs and NMOs form monophyletic groups whereas FMOs form a paraphyletic group of genes including the so-called YUCCAs.
4. An evolutionary model, in which FMOs, NMOs and BVMOs genes were already present in LUCA, is proposed here.

## Materials & Methods

### Data retrieval and multiple sequence alignments

To carry out a survey for the presence of BVMOs encoding sequences across species, a deep *in silico* homology search was performed using genomic (refseq genomic, gss, wgs), transcriptomic (refseq rna, ESTs) and protein (refseq protein) databases at NCBI. The search was conducted as an iterative process in each life domain as follows; (i) BLASTp: PHI-BLAST using as a query PAMO sequence (Q47PU3.1) and the two previously described BVMO canonical motifs: [A/G]GxWxxxx[F/Y]P[G/M]xxxD [24] and FxGxxxHxxxW[P/D] [22], (ii) BLASTp using as a query each sequence of the proteins identified in the previous step and (iii) tBLASTn in nucleotide databases using as a query each sequence of the proteins identified in the two previous steps. The complete set of sequences is available in S1 Table. Multiple sequence alignments (MSA) were performed using the online version of MAFFT 7 program and the BLOSUM 62 scoring matrix [55]. For alignment visualization MEGA 6.0 software was employed. Additionally, a search was conducted using HMMER (http://hmmer.janelia.org/) with the hmmsearch tool (protein alignment *vs*. protein sequence database) and the UniProtKB database [56]. However no additional sequences were collected. This analysis provided the HMM logos (S2 Fig) for the MSA used as input. All the retrieved sequences were curated by confirming the presence of the two BVMOs fingerprints [22, 24] and the two Rossmann motifs (GxGxx[G/A]). The MSA was edited excluding N- and C-terminal regions flanking the two Rossmann motifs. A dataset of 121 BVMOs was obtained (S1 Dataset).

To construct the dataset of FMOs, YUCCAs and NMOs, BLASTp searches in protein databases of Archaea, Bacteria and Eukarya domains were performed. For FMOs, sequences of FMO 3 from *Homo sapiens* (NP_008825.4), FMO from Rabbit (AAB19844.1) and FMO GS-OX1 from *Arabidopsis thaliana* (NP_176761.1) were employed as queries in different searches. Besides the FMO typifying motif FxGxxxHxxxY[K/R] [42] was used to revise the retrieved sequences. For NMOs, the sequences of L-ornithine N5-oxygenase SidA from *Aspergillus fumigatus* (XP_755103.1) and the alcaligin biosynthesis enzyme from *Bordetella bronchiseptica* (Q44740.2) were used as queries. The YUCCAs sequences were obtained from the literature [15]. The complete set of sequences is available in S2 Table. MSAs were performed for each group of flavoproteins to analyze the presence of conserved motifs, meaning the two Rossmann motifs for all “Class B” monooxygenases and the specific fingerprint in the case of FMOs. The alignment was edited excluding N- and C-terminal regions flanking the two Rossmann fingerprints. A complete alignment, including BVMOs, FMOs, YUCCAs and NMOs sequences, was constructed (S2 Dataset). Besides, a MSA including all collected “Class B” flavin monooxygenases (BVMOs, NMOs, FMOs and YUCCAs) plus a group of “Class A” flavin monooxygenases (to be used as an outgroup) was constructed (S3 Dataset). “Class A” monooxygenases were obtained from previous reports [11].

### Construction of phylogenetic trees

Two independent approaches were used to construct the phylogenetic trees of BVMOs and of “Class B” monooxygenases. The first approach was using the Maximum likelihood (ML) method. For this, MSAs previously constructed were manually edited to eliminate gaps and the best fit model parameters were calculated employing ProtTest 3.4 version [57]. To build the tree PhyML 3.0 on-line tool was used [58]. To estimate the robustness of the phylogenetic inference 100 bootstraps were run. To visualize and edit the consensus tree MEGA 6.0 software was used.

The other approach consisted in employing Bayesian inference method. The edited MSAs employed for ML trees, were used as input to build the tree using Mr. Bayes 3.2 software [59]. A mixed amino acid substitution model was set up, 4 gamma categories and a proportion of invariable sites were considered. The analyses were concluded after 1,000,000 generations when the split frequency was < 0.02. To visualize and edit the tree, FigTree 1.4.2 software was used.

### Genomic context analyses

To analyze the genomic context of selected BVMOs (*e*.*g*: BVMOs detected in *Pantholops hodgsonii*, ACMO from *Gordonia* sp. Odio1 and Odio3 from *Oikopleura doica*, etc.) whole contigs were downloaded. Initially these fragments were subjected to BLASTn searches in reference genomic sequences (refseq_genome) and genomic survey sequences (gss) databases. Then, individual genes upstream and downstream from the BVMO open reading frame (ORF) were analyzed by tBLASTn and the retrieved sequences were subjected to reciprocal tBLASTn in order to determine possible orthologous/xenologous sequences. Also, the contigs were subjected to progressive alignment using MAUVE software [60] with comparison purposes.

### PCR experiments

PCR experiments were conducted to confirm the presence of BVMO encoding sequences in selected organisms. Genomic DNA of *Hordeum vulgare* subsp. *vulgare* was obtained from Instituto Nacional de Tecnología Agropecuaria (INTA-Castelar). Genomic DNA from *Emiliania huxleyi* was obtained from Provasoli-Guillard National Center for Culture of Marine Phytoplankton strain CCMP #373. Genomic DNA from *Aspergillus flavus* was purified from a growing culture, employing the Wizard Genomic DNA extraction Kit (Promega). Primers were designed to amplify each BVMO sequence and also housekeeping genes (S3 Table). Fragments were amplified using *Taq*-UBA DNA polymerase (gifted by Dr. Mauro Morgenfeld). PCR conditions were: initial denaturation for 2 min at 94°C, followed by 35 cycles of denaturation (30s at 94°C), annealing (30s at Ta°C) and extension (30-45s at 72°C), final extension of 10 min at 72°C.

## Supporting Information

S1 Dataset BVMOs multiple sequence alignment. (FASTA)

S2 Dataset BVMOs, FMOs, YUCCAs and NMOs multiple sequence alignment. (FASTA)

S3 Dataset BVMOs, FMOs, YUCCAs, NMOs and “Class A” flavin monooxygenases multiple sequence alignment. (FASTA)

S1 Fig Distribution of sequence identities among the BVMOs dataset. The histogram displays the ranges of sequence identity *vs*. the amount (as percentage) of sequences. The identity matrix was constructed employing CLUSTALW program and the BLOSUM62 scoring matrix.
(TIF)

S2 Fig HMM logos of BVMOs sequences. The diagram was obtained from S1 Dataset by performing an analysis employing HMMER tool (available at http://hmmer.janelia.org/), as depicted in Materials & Methods section. The two Rossmann motifs (GxGxx[G/A]) and the two BVMO fingerprints ([A/G]GxWxxxx[F/Y]P[G/M]xxxD and FxGxxxHxxxW[P/D]) are highlighted. Arrow indicates the conserved Thr residue detected by Rebehmed *et al* [23].
(TIF)

S3 Fig PCR experiments to detect the BVMO gene in *Hordeum vulgare*. (PDF)

S4 Fig PCR experiments to detect BVMO genes in *Emiliania huxleyi*. (PDF)

S5 Fig Phylogenetic analysis of BVMOs protein-family employing Bayesian method. (PDF)

S6 Fig Rooted phylogenetic tree of “Class B” flavin monooxygenases employing Bayesian method. The tree was constructed by using the Bayesian method, employing the alignment constructed with MAFFT 7 on-line tool. Evolutionary analyses were conducted using Mr.Bayes 3.2 software. Posterior probabilities values are shown next to the branches. Colored branches display: BVMOs (yellow), NMOs (blue) and FMOs (black). As outgroup, hydroxylases belonging to “Class A” flavin monooxygenases were employed (orange).
(PDF)

S7 Fig Phylogenetic analysis of selected “Class B” monooxygenases by Maximum Likelihood (ML) method. The tree was constructed by using the ML method, employing the alignment constructed with MAFFT 7 on-line tool and the best model parameters calculated with ProTest 3.4. Evolutionary analyses were conducted in PhyML 3.0 on-line server. Bootstrap values (> 45) are shown next to the branches. Colored branches show: BVMOs (yellow), NMOs (blue), FMOs & YUCCAs (black). Colored triangles display the source of BVMO encoding genes as follows: Fungi (black), Bacteria (orange), Green plants (dark green), Rhodophyta (light green), Metazoa (red), Haptophyta (purple), Archaea (blue), Chlorophyta (light blue).
(PDF)

S1 File Detection of contaminating sequences in *Pantholops hodgsonii* genome draft. (PDF)

S2 File Analyses of *Adineta vaga* genes proposed to be acquired by HGT. (PDF)

S3 File Analysis of the phylogenetic distribution of BVMOs and its substrate specificity. (PDF)

S4 File Conserved domain architecture analysis of identified BVMO sequences. (PDF)

S1 Table BVMO sequences employed in this work. All BVMOs protein sequences employed in this work are depicted. Colors are used to display the origin of each sequence, as follows: Bacteria (white), Fungi (grey), Green plants and Rhodophyta (green), Haptophyta (light purple), Metazoa (orange) and Archaea (blue). Sequences marked as (*) have been previously characterized. Sequences marked as (#) have been previously named as AFL838, AFL619, AFL210 and AFL456, respectively [20]. Sequences without marks are reported here for the first time as putative BVMOs. Most of these sequences are derived from automated annotation of genomic data.
(PDF)

S2 Table Other “Class B” flavin monooxygenases encoding sequences employed in this work. (PDF)

S3 Table Primer sequences and PCR conditions. (PDF)
