# Supplementary figures and images for: The Origin and Evolution of Baeyer—Villiger Monooxygenases (BVMOs): An Ancestral Family of Flavin Monooxygenases
Source: PLoS One. 2015 Jul 10;10(7):e0132689. doi: 10.1371/journal.pone.0132689 (PMC4498894; doi:10.1371/journal.pone.0132689)

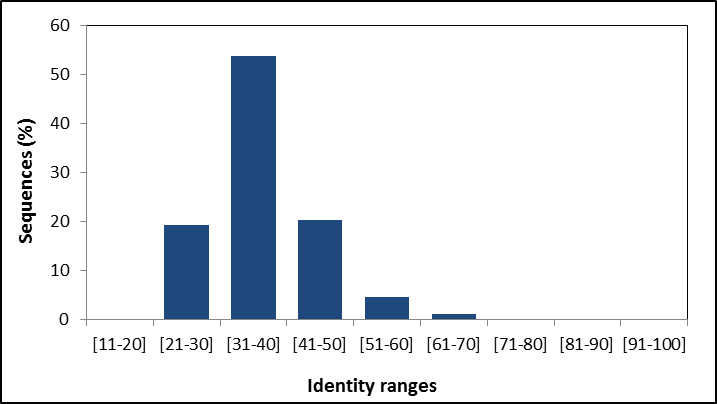

Supplement: S1 Fig — The histogram displays the ranges of sequence identity vs. the amount (as percentage) of sequences. The identity matrix was constructed employing CLUSTALW program and the BLOSUM62 scoring matrix. (TIF) [file pone.0132689.s004.tif]

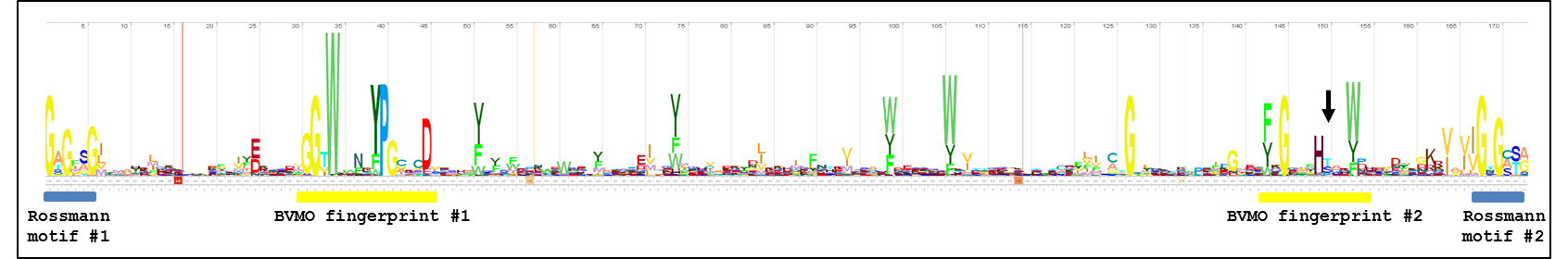

Supplement: S2 Fig — The diagram was obtained from S1 Dataset by performing an analysis employing HMMER tool (available at http://hmmer.janelia.org/), as depicted in Materials & Methods section. The two Rossmann motifs (GxGxx[G/A]) and the two BVMO fingerprints ([A/G]GxWxxxx[F/Y]P[G/M]xxxD and FxGxxxHxxxW[P/D]) are highlighted. Arrow indicates the conserved Thr residue detected by Rebehmed et al [23]. (TIF) [file pone.0132689.s005.tif]

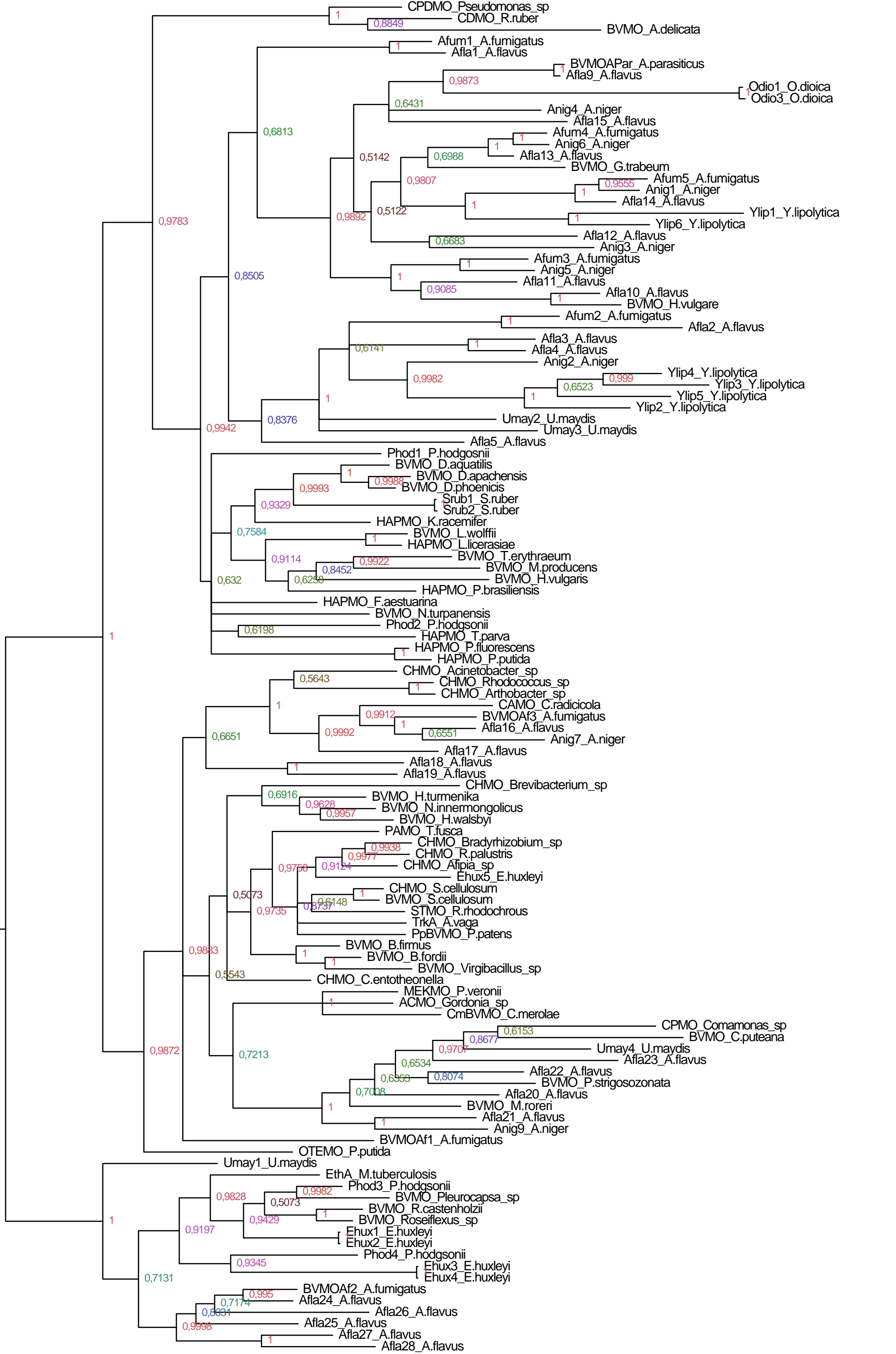

Supplement: S5 Fig — (PDF) [file pone.0132689.s008.pdf]

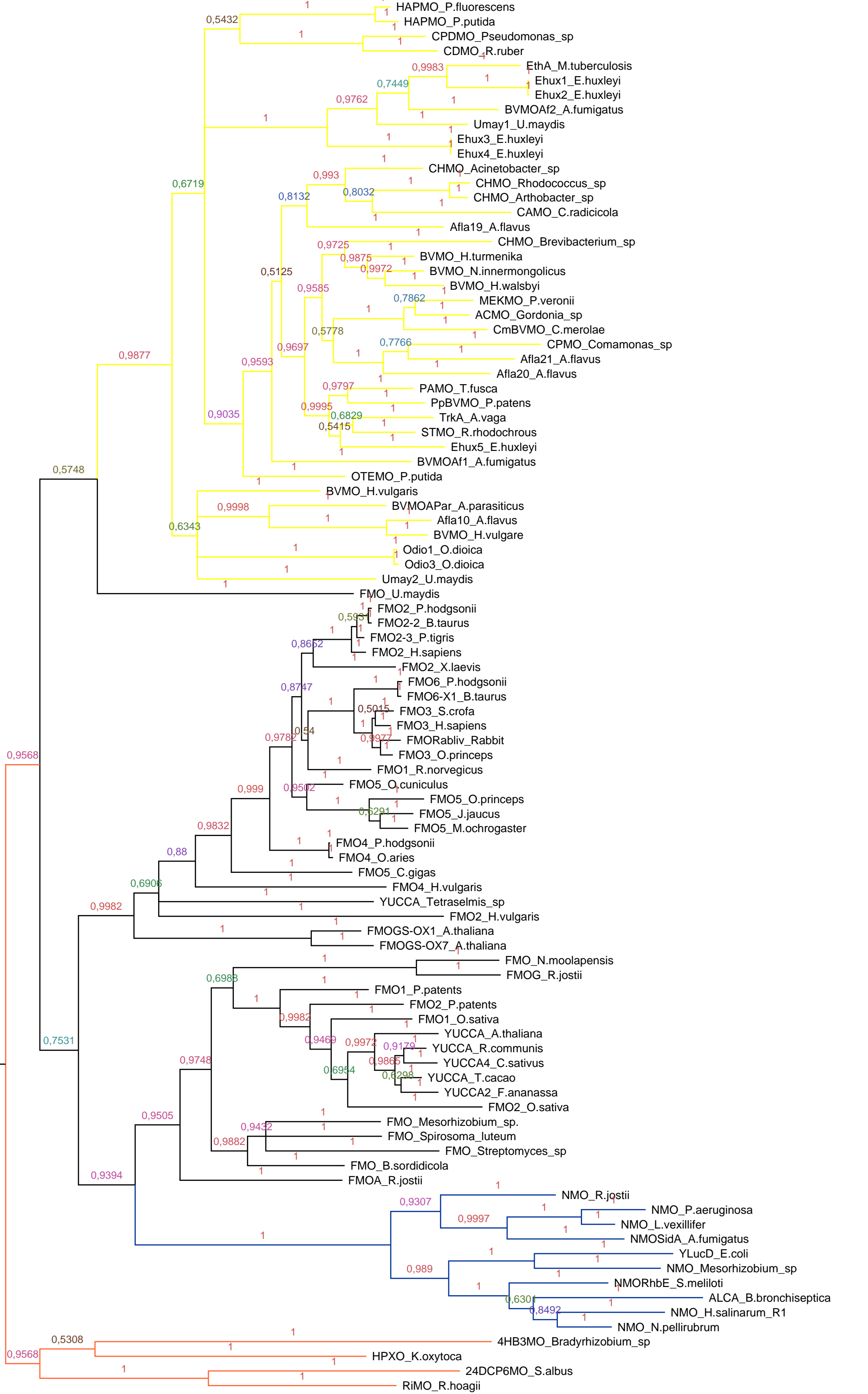

Supplement: S6 Fig — The tree was constructed by using the Bayesian method, employing the alignment constructed with MAFFT 7 on-line tool. Evolutionary analyses were conducted using Mr.Bayes 3.2 software. Posterior probabilities values are shown next to the branches. Colored branches display: BVMOs (yellow), NMOs (blue) and FMOs (black). As outgroup, hydroxylases belonging to “Class A” flavin monooxygenases were employed (orange). (PDF) [file pone.0132689.s009.pdf]

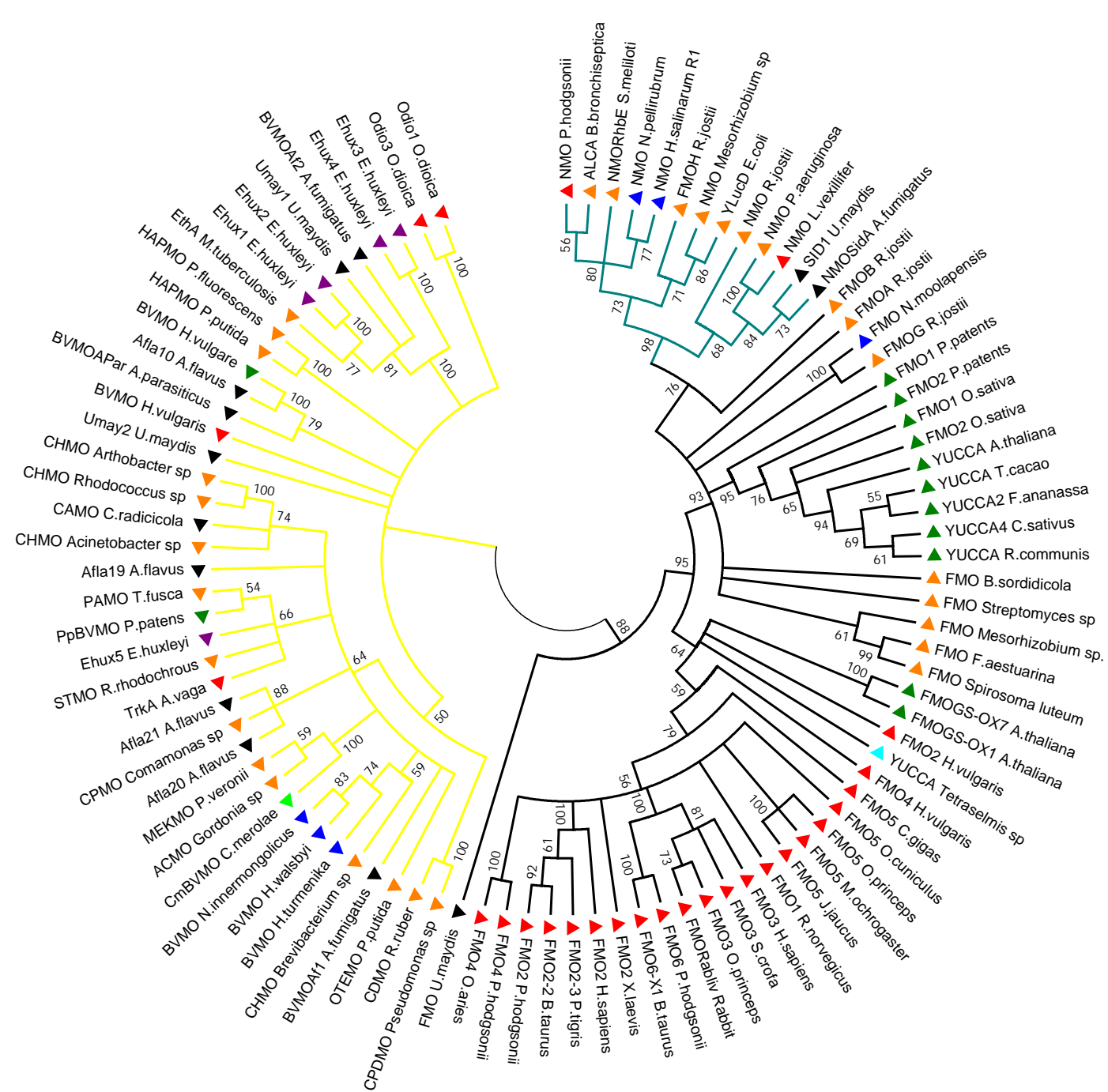

Supplement: S7 Fig — The tree was constructed by using the ML method, employing the alignment constructed with MAFFT 7 on-line tool and the best model parameters calculated with ProTest 3.4. Evolutionary analyses were conducted in PhyML 3.0 on-line server. Bootstrap values (> 45) are shown next to the branches. Colored branches show: BVMOs (yellow), NMOs (blue), FMOs & YUCCAs (black). Colored triangles display the source of BVMO encoding genes as follows: Fungi (black), Bacteria (orange), Green plants (dark green), Rhodophyta (light green), Metazoa (red), Haptophyta (purple), Archaea (blue), Chlorophyta (light blue). (PDF) [file pone.0132689.s010.pdf]
