# Supplementary material for: The Origin and Evolution of Baeyer—Villiger Monooxygenases (BVMOs): An Ancestral Family of Flavin Monooxygenases
Source: PLoS One. 2015 Jul 10;10(7):e0132689. doi: 10.1371/journal.pone.0132689 (PMC4498894; doi:10.1371/journal.pone.0132689)
Supplement: S3 Fig — (PDF) [file pone.0132689.s006.pdf]

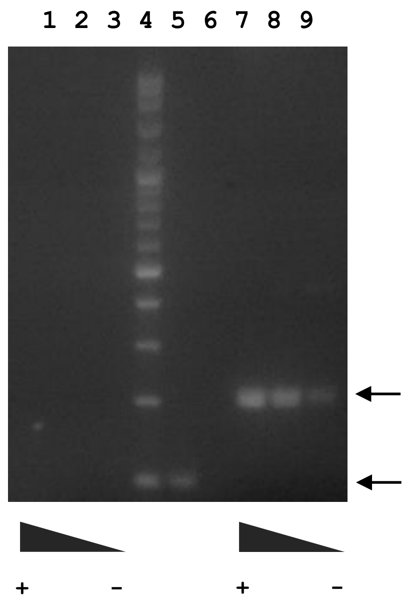

Lanes 1-3: Amplification of 107 bp fragment of BVMO gene from barley, employing decreasing amounts of genomic DNA (1 μg/μl; 0.5 μg/μl; 0.1μg/μl). Lane 4: 100 bp plus ladder (genbiotech). Lane 5: Amplification of 107 bp fragment BVMO Afla10 in *A. flavus* employing 0.1 μg/μl of genomic DNA. Lane 6: negative control. Lanes 7-9 Amplification of a 205 bp fragment of the housekeeping gene α-tubulin from barley, employing decreasing amounts of genomic DNA (1 μg/μl; 0.5 μg/μl; 0.1μg/μl).
