# Supplementary material for: The Origin and Evolution of Baeyer—Villiger Monooxygenases (BVMOs): An Ancestral Family of Flavin Monooxygenases
Source: PLoS One. 2015 Jul 10;10(7):e0132689. doi: 10.1371/journal.pone.0132689 (PMC4498894; doi:10.1371/journal.pone.0132689)
Supplement: S4 Fig — (PDF) [file pone.0132689.s007.pdf]

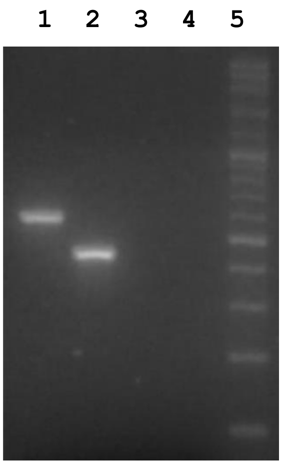

Lane 1: Amplification of 588 bp fragment of *Ehux1*/*Ehux2*. Lane 2: Amplification of 441 bp fragment of *Ehux3*/*Ehux4*. Lane 3: Amplification of *Ehux5* fragment. Lane 4: negative control. Lane 5: 100 bp plus ladder (genbiotech).

Further gradient PCR (Ta°C= 47°C-53°C) experiments were conducted to verify the presence of *Ehux5* gene. No positive amplification was observed at any of the tested conditions.
