# Supplementary material for: The Origin and Evolution of Baeyer—Villiger Monooxygenases (BVMOs): An Ancestral Family of Flavin Monooxygenases
Source: PLoS One. 2015 Jul 10;10(7):e0132689. doi: 10.1371/journal.pone.0132689 (PMC4498894; doi:10.1371/journal.pone.0132689)
Supplement: S1 File — (PDF) [file pone.0132689.s011.pdf]

# Detection of contaminating sequences in *Pantholops hodgsonii* genome draft

A puzzling observation was the finding of *BVMO* encoding sequences in the draft genome of a mammal, the Tibetan antelope *P. hodgsonii* [1]. This animal belongs to the family bovidae although its precise phylogenetic location remains debated [2, 3]. This is the only mammal where *BVMOs* were detected, finding 4 different paralogs. Intriguingly, when other complete genomes of the bovidae family (*Bos taurus*, *Bos indicus*, *Capra hircus*, *Ovis aries*, *Sus crofa* and *Ochotona princeps*) were investigated for the presence of BVMOs, no positive hits were found.

In order to revise the genomic context of these genes, a homology analysis was performed by BLASTp and tBLASTn followed by multiple sequence alignments. The Phod1-4 protein sequences (XP_005978382.1, XP_005978142.1, XP_005978883.1 and XP_005973192.1) displayed high identity (61-98%) with bacterial annotated proteins (WP_004720774, WP_031447074, WP_004623235 and WP_010112128, respectively). When the complete contigs were analyzed by performing BLASTn, two out of the four were virtually identical to genome fragments from bacteria. The contig containing *Phod1* sequence (NW_005815101.1) showed 99% identity across 171,282 nt with the genome from *Acinetobacter guillouiae* strain NIPH 991 (NZ_KB849456.1). On the other hand, the contig containing *Phod4* sequence (NW_005813336.1) displayed 91% identity, in 34,626 nt, with *Acinetobacter bereziniae* CIP 70.12 genome (NZ_KB849756.1). These observations were confirmed by progressive alignment analyses. When further analyses of *P. hodgsonii* draft genome were carried through, several similar cases were observed for other annotated contigs. These observations are in line with a recent report indicating the presence of bacterial contaminants in *P. hodgsonii* draft genome sequence [4].

These results indicate that the contigs containing BVMOs in *P. hodgsonii* come from a bacterial contamination somewhere in the pipeline of the sequencing project. Thus the presence

of BVMOs in mammal genomes results an artifact (a false positive) and not a true positive finding.


1. Ge RL, Cai Q, Shen YY, San A, Ma L, Zhang Y, et al. Draft genome sequence of the Tibetan antelope. Nat Commun. 2013;4:1858. Epub 2013/05/16. doi: ncomms2860 [pii]
10.1038/ncomms2860. PubMed PMID: 23673643; PubMed Central PMCID: PMC3674232.
2. Shafer AB, Hall JC. Placing the mountain goat: a total evidence approach to testing alternative hypotheses. Mol Phylogenet Evol. 2010;55(1):18-25. Epub 2010/01/26. doi: S1055-7903(10)00017-5 [pii]
10.1016/j.ympev.2010.01.015. PubMed PMID: 20097296.
3. Yang C, Xiang C, Qi W, Xia S, Tu F, Zhang X, et al. Phylogenetic analyses and improved resolution of the family Bovidae based on complete mitochondrial genomes. Biochemical Systematics and Ecology. 2013;48(0):136-43. doi: http://dx.doi.org/10.1016/j.bse.2012.12.005.
4. Laurence M, Hatzis C, Brash DE. Common contaminants in next-generation sequencing that hinder discovery of low-abundance microbes. PLoS One. 2014;9(5):e97876. Epub 2014/05/20. doi: 10.1371/journal.pone.0097876
PONE-D-14-10042 [pii]. PubMed PMID: 24837716; PubMed Central PMCID: PMC4023998.
