# Supplementary material for: The Origin and Evolution of Baeyer—Villiger Monooxygenases (BVMOs): An Ancestral Family of Flavin Monooxygenases
Source: PLoS One. 2015 Jul 10;10(7):e0132689. doi: 10.1371/journal.pone.0132689 (PMC4498894; doi:10.1371/journal.pone.0132689)
Supplement: S2 File — (PDF) [file pone.0132689.s012.pdf]

Analyses of *Adineta vaga* genes proposed to be acquired by HGT

The *BVMO* encoding sequence found in *A.vaga* (*TrkA*; Gene Id: EU643481.1 (21177-22838); Prot. Id: ACD54725.1) has been previously described to be laterally-transferred from a bacterial genome to the rotifer. As it was discussed in in our article, we have found evidence suggesting an alternative hypothesis to the HGT by the gain and loss of paralogous genes. This alternative explains more parsimoniously the presence of this BVMO in *A. vaga* genome.

Considering this alternative scenario, we further analyzed other possible horizontally-transferred genes in *Adineta vaga*. For this BLASTp analyses of the 11 genes with the highest AI, reported by Gladyshev *et al* [1], were performed (see table below). Among these sequences, we found three genes (ACD54724.1, ACD54788.1 and ACD54765.1) that at least have one paralog in *A. vaga*, with amino acids sequence identities ranging 35-42%. If these genes would have been acquired by HGT, this event should have taken place in an earlier ancestor. An alternative explanation could be the occurrence of double horizontal gene transfer and then independent gene evolution. However, this hypothesis is clearly less-parsimonious. Other four genes (ACD54778.1, ACD54781.1, ACD54811.1 and ACD54602.1) were found to have homologs in metazoans with hit values (*i.e.*: identity and E-value) very close to those from bacteria and/or fungi. Once again, it was observed that the retrieved sequences have been deposited after 2010. Only two sequences (ACD54637.1, ACD54790.1) remained with no metazoan homologs, thus being possible candidates to have been acquired by HGT. This analysis supports the notion that the contribution of HGT to eukaryotic genomes evolution may be largely overestimated as a consequence of the limited genomic or transcriptomic data available in databases and inadequate sequence analyses, since homology searching does not constitute enough evidence to propose the occurrence of HGT [2].

Representative CDS of *Adineta vaga* proposed to be acquired by HGT

Gene ID	Contig Id	AI	Best Hit	Taxonomy	Id(C)*	E-value	Best Hit Metazoa	Id(C)	E-value	Date
**ACD54637.1 (AV10027)**	Av212_A	460	WP_026896859.1 *Pedobacter oryzae*	Bacteria	73(96)	0.0	No hit			
**ACD54724.1 (AV10001)**	Av110_A	460	ACD54728.1 *Adineta vaga*	Metazoa	94(98)	0.0	ACD54728.1 *Adineta vaga*	94(98)	0.0	2008
			ACD54765.1 *Adineta vaga*	Metazoa	41(74)	0.0	ACD54765.1 *Adineta vaga*	41(74)	0.0	2008
			WP_006529541.1 *Gloeocapsa* sp.	Bacteria	34(84)	0.0	XP_004532053.1 *Ceratitis capitata*	36(37)	2e-179	2013
**ACD54778.1 (AV10134)**	161F107	400	EMR88727.1 *Botrytis cinerea*	Fungi	62(96)	0.0	XP_003383373.1 *Amphimedon queenslandica*	28(94)	5e-32	2011
**ACD54725.1 AV10002**	Av110_A	379	ETR78797.1 *Afipia* sp.	Bacteria	55(96)	0.0	CBY20261.1 *Oikopleura dioica*	25(89)	2e-38	2010
							XP_002164118.2 *Hydra vulgaris*	36(74)	9e-35	2013
**ACD54781.1 (PR10002)**	182F10	327	WP_019942145.1 *Dyadobacter beijingensis*	Bacteria	68(96)	0.0	XP_005366850.1 *Microtus ochrogaster*	25(64)	3e-09	2013
**ACD54788.1 (PR10010)**	182F10	310	WP_020740068.1 *Sorangium cellulosum*	Bacteria	33(97)	7e-136	ACD54642.1 *Adineta vaga*	35(97)	8e-43	2008
**ACD54790.1 (PR10012)**	182J17	246	XP_002671477.1 *Naegleria gruberi*	Bacteria	49(93)	0.0	No hit			
**ACD54765.1 (AV10121)**	99O7	237	ACD54724.1 *Adineta vaga*	Metazoa	41(98)	0.0	ACD54724.1 *Adineta vaga*	41(98)	0.0	2008
			ACD54728.1 *Adineta vaga*	Metazoa	42(98)	0.0	ACD54728.1 *Adineta vaga*	42(98)	0.0	2008
			WP_005264033.1 *Rhodococcus opacus*	Bacteria	30(62)	1e-115				
**ACD54811.1 (AV10153)**	210B3	212	XP_001549360.1 *Botrytis cinerea*	Fungi	50(81)	1e-129	AIB04028.1 *Apolygus lucorum*	28(58)	7e-17	2014
**ACD54602.1 (AV10042)**	Av240B	199	ACD54583.1 *Adineta vaga*	Metazoa	99(100)	0.0	ACD54583.1 *Adineta vaga*	99(100)	0.0	2008
			WP_026083861.1 *Pseudomonas* sp.	Bacteria	58(95)	2e-118	XP_004532296.1 *Ceratitis capitata*	30(41)	3e-08	2013

The genes with highest alien index (AI) reported by Gladyshev *et al*, were analyzed by BLASTp searching. Headline columns in blue show BLASTp searches against non-redundant protein sequences (nr) of any organism. Headline in pink show searches against metazoan non-redundant protein sequences (nr). The BVMO TrkA row is displayed in yellow. Best hit is defined as the hit with highest identity and E-value closest to zero. Annotation date for best metazoan hit is provided.

*Id(C)= identity % (total coverage %).

1. Gladyshev EA, Meselson M, Arkhipova IR. Massive horizontal gene transfer in bdelloid rotifers. Science. 2008;320(5880):1210-3. Epub 2008/05/31. doi: 320/5880/1210 [pii]
10.1126/science.1156407. PubMed PMID: 18511688.
2. Snel B, Bork P, Huynen MA. Genome phylogeny based on gene content. Nat Genet. 1999;21(1):108-10. Epub 1999/01/23. doi: 10.1038/5052. PubMed PMID: 9916801.
