# Supplementary material for: The Origin and Evolution of Baeyer—Villiger Monooxygenases (BVMOs): An Ancestral Family of Flavin Monooxygenases
Source: PLoS One. 2015 Jul 10;10(7):e0132689. doi: 10.1371/journal.pone.0132689 (PMC4498894; doi:10.1371/journal.pone.0132689)
Supplement: S3 File — (PDF) [file pone.0132689.s013.pdf]

Analysis of the phylogenetic distribution of BVMOs and its substrate specificity

In the literature it has been largely stated that BVMOs cluster together according to its substrate specificity [1, 2]. To test this hypothesis, we analyzed the distribution of the already characterized BVMOs in our phylogenetic tree (Fig. 2 in the manuscript). To do this, we generated a tree in which branches displaying: (i) BVMO encoding sequences identified for the first time in this work and (ii) BVMO sequences with non-natural substrates identified, are collapsed (Fig. below). Branches were colored according to BVMOs substrate specificity and the structure of the substrate was placed on the right (a full list of BVMO sequences with its natural substrates and the specific references is provided in a table below).

This analysis suggests that BVMOs do not cluster according to its substrate specificity. However it is important to highlight that BVMOs have been deeply characterized using non-natural substrates, mainly with biotechnological purposes. Therefore, there is very limited information about their physiological substrates.

---

1. Mihovilovic MD, Rudroff F, Grotzl B, Kapitan P, Snajdrova R, Rydz J, et al. Family clustering of Baeyer-Villiger monooxygenases based on protein sequence and stereopreference. Angew Chem Int Ed Engl. 2005;44(23):3609-13. Epub 2005/04/30. doi: 10.1002/anie.200462964. PubMed PMID: 15861400.

2. Fink MJ, Fischer TC, Rudroff F, Dudek H, Fraaije MW, Mihovilovic MD. Extensive substrate profiling of cyclopentadecanone monooxygenase as Baeyer–Villiger biocatalyst reveals novel regiodivergent oxidations. Journal of Molecular Catalysis B: Enzymatic. 2011;73(1–4):9-16. doi: http://dx.doi.org/10.1016/j.molcatb.2011.07.003.

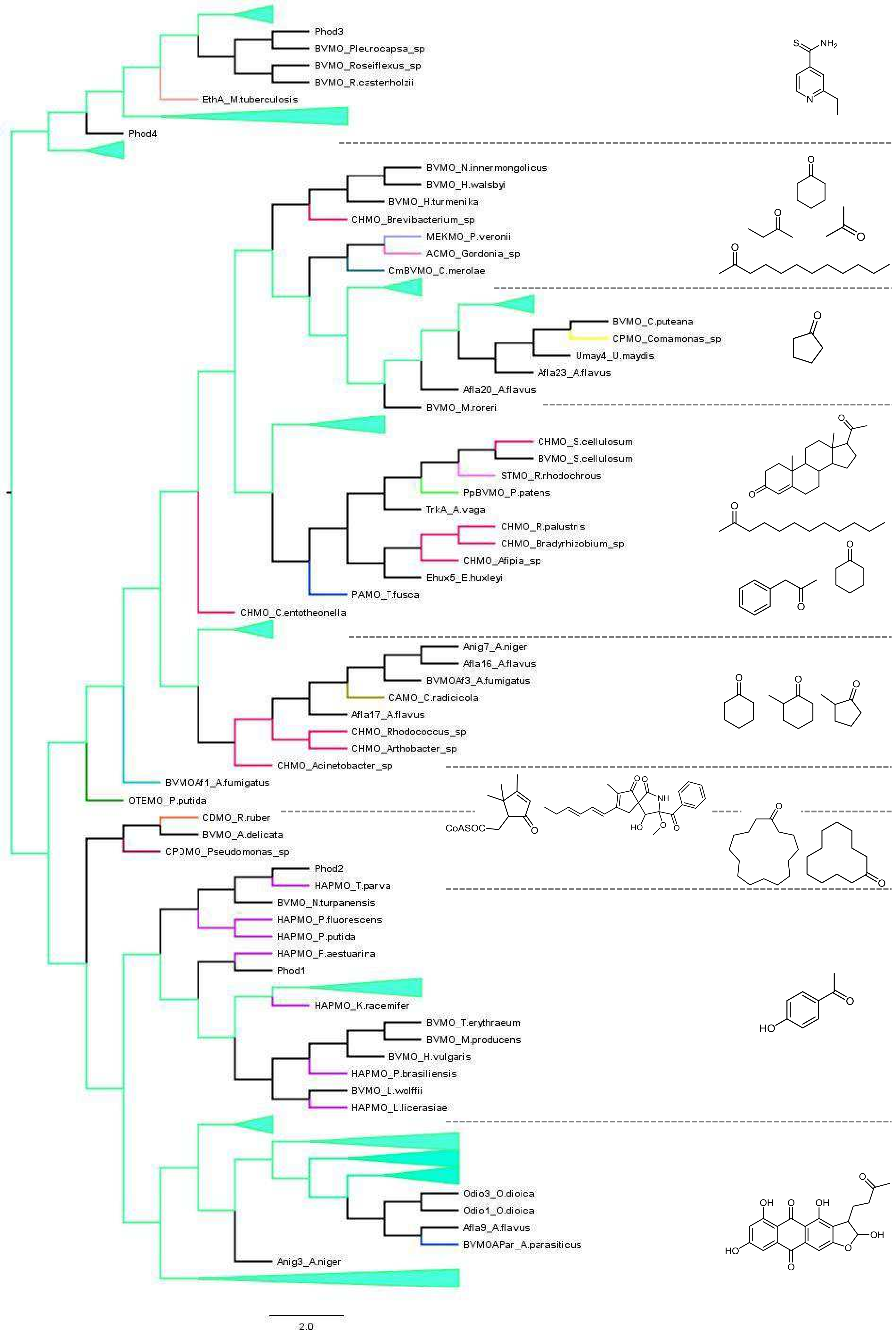
Phod3
BVMO_Pleurocapsa_sp
BVMO_Roseiflexus_sp
BVMO_R.castenholzii
EthA_M.tuberculosis
Phod4
BVMO_N.innermongolicus
BVMO_H.walsbyi
BVMO_H.turmenika
CHMO_Brevibacterium_sp
MEKMO_P.veronii
ACMO_Gordonia_sp
CmBVMO_C.merolae
BVMO_C.puteana
CPMO_Comamonas_sp
Umay4_U.maydis
Afla23_A.flavus
Afla20_A.flavus
BVMO_M.roreri
CHMO_S.cellulosum
BVMO_S.cellulosum
STMO_R.rhodochrous
PpBVMO_P.patens
TrkA_A.vaga
CHMO_R.palustris
CHMO_Bradyrhizobium_sp
CHMO_Afipia_sp
Ehux5_E.huxleyi
PAMO_T.fusca
CHMO_C.entotheonella
Anig7_A.niger
Afla16_A.flavus
BVMOAf3_A.fumigatus
CAMO_C.radicicola
Afla17_A.flavus
CHMO_Rhodococcus_sp
CHMO_Arthobacter_sp
CHMO_Acinetobacter_sp
BVMOAf1_A.fumigatus
OTEMO_P.putida
CDMO_R.ruber
BVMO_A.delicata
CPDMO_Pseudomonas_sp
Phod2
HAPMO_T.parva
BVMO_N.turpanensis
HAPMO_P.fluorescens
HAPMO_P.putida
HAPMO_F.aestuarina
Phod1
HAPMO_K.racemifer
BVMO_T.erythraeum
BVMO_M.producens
BVMO_H.vulgaris
HAPMO_P.brasiliensis
BVMO_L.wolffii
HAPMO_L.licerasiae
Odio3_O.dioica
Odio1_O.dioica
Afla9_A.flavus
BVMOAPar_A.parasiticus
Anig3_A.niger
2.0
CoASOC
HO

BVMO	Organism	Substrate	Reference
**EthA**	*Mycobacterium tuberculosis*	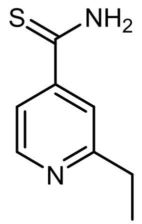 Ethionamide	[1]
**CHMO**	*Acinetobacter* NCIMB 9871	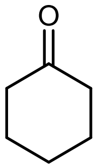 Cyclohexanone	[2]
**MEKMO**	*Pseudomonas veronii* MEK700	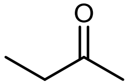 Butanone	[3]
**ACMO**	*Gordonia sp* TY-5	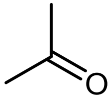 Acetone	[4]
**CmBVMO**	*Cyanidioschyzon merolae*	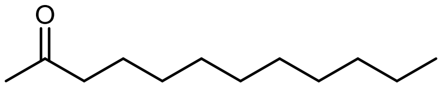 Dodecanone	[5]
**CPMO**	*Comamonas sp* NCIMB 9872	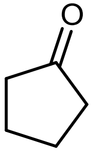 Cyclopentanone	[6]

**STMO**	*Rhodococcus rhodochrous*	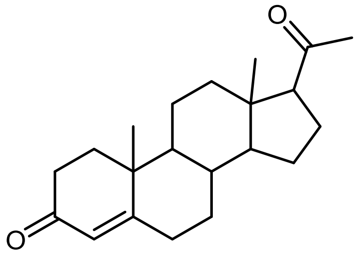 Dehydro-*epi*-androsterone	[7]
**PpBVMO**	*Physcomitrella patens*	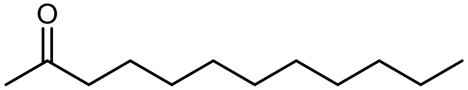 Dodecanone	[5]
**PAMO**	*Thermobifida fusca*	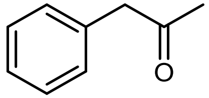 Phenylacetone	[8]
**BVMO$_{Af1}$**	*Aspergillus fumigatus*	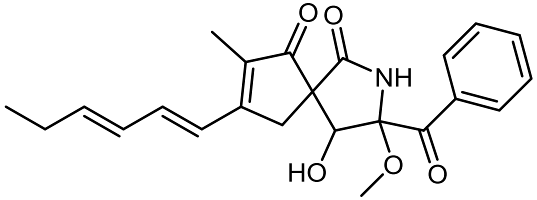 Deepoxy-synerazole	[9]
**CAMO**	*Cylindrocarpon radicicola*	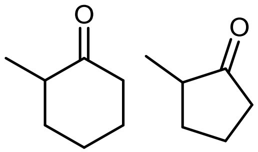 2-methylcyclohexanone, 2-methylcyclopentanone	[10]

**OTEMO**	*Pseudomonas putida* ATCC 17453	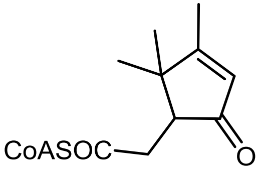<br>2-oxo-Delta(3)-4,5,5-trimethylcyclopentenylacetyl-coenzyme A	[11]
**CDMO**	*Rhodococcus ruber* SC1	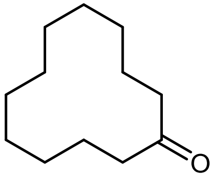<br>Cyclododecanone	[12]
**CPDMO**	*Pseudomonas sp*	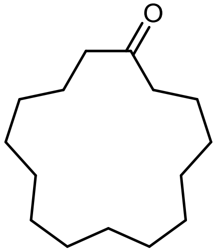<br>Cyclopentadecanone	[13]
**HAPMO**	*Pseudomonas fluoresces* ACB	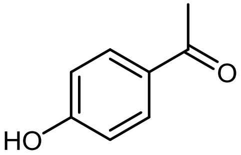<br>4-hydroxyacetophenone	[14]

**BVMO$_{Apar}$**	*Aspergillus parasiticus*	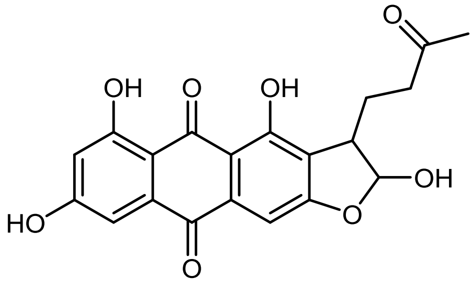<br>Hydroxyversicolorone	[15]

References

1. Fraaije MW, Kamerbeek NM, Heidekamp AJ, Fortin R, Janssen DB. The prodrug activator EtaA from Mycobacterium tuberculosis is a Baeyer-Villiger monooxygenase. J Biol Chem. 2004;279(5):3354-60. Epub 2003/11/12. doi: 10.1074/jbc.M307770200
M307770200 [pii]. PubMed PMID: 14610090.

2. Trudgill PW. Cyclohexanone 1,2-monooxygenase from Acinetobacter NCIMB 9871. Methods Enzymol. 1990;188:70-7. PubMed PMID: 2177829.

3. Volker A, Kirschner A, Bornscheuer UT, Altenbuchner J. Functional expression, purification, and characterization of the recombinant Baeyer-Villiger monooxygenase MekA from Pseudomonas veronii MEK700. Appl Microbiol Biotechnol. 2008;77(6):1251-60. doi: 10.1007/s00253-007-1264-6. PubMed PMID: 18034235.

4. Kotani T, Yurimoto H, Kato N, Sakai Y. Novel acetone metabolism in a propane-utilizing bacterium, Gordonia sp. strain TY-5. J Bacteriol. 2007;189(3):886-93. doi: 10.1128/JB.01054-06. PubMed PMID: 17071761; PubMed Central PMCID: PMC1797311.

5. Beneventi E, Niero M, Motterle R, Fraaije M, Bergantino E. Discovery of Baeyer–Villiger monooxygenases from photosynthetic eukaryotes. Journal of Molecular Catalysis B: Enzymatic. 2013;98(0):145-54. doi: http://dx.doi.org/10.1016/j.molcatb.2013.10.006.

6. Iwaki H, Hasegawa Y, Wang S, Kayser MM, Lau PC. Cloning and characterization of a gene cluster involved in cyclopentanol metabolism in Comamonas sp. strain NCIMB 9872 and biotransformations effected by Escherichia coli-expressed cyclopentanone 1,2-monooxygenase. Appl Environ Microbiol. 2002;68(11):5671-84. PubMed PMID: 12406764; PubMed Central PMCID: PMC129933.

7. Franceschini S, van Beek HL, Pennetta A, Martinoli C, Fraaije MW, Mattevi A. Exploring the structural basis of substrate preferences in Baeyer-Villiger monooxygenases: insight from steroid monooxygenase. J Biol Chem. 2012;287(27):22626-34. doi: 10.1074/jbc.M112.372177. PubMed PMID: 22605340; PubMed Central PMCID: PMC3391149.

8. Fraaije MW, Wu J, Heuts DP, van Hellemond EW, Spelberg JH, Janssen DB. Discovery of a thermostable Baeyer-Villiger monooxygenase by genome mining. Appl Microbiol Biotechnol. 2005;66(4):393-400. doi: 10.1007/s00253-004-1749-5. PubMed PMID: 15599520.

9. Wiemann P, Guo CJ, Palmer JM, Sekonyela R, Wang CC, Keller NP. Prototype of an intertwined secondary-metabolite supercluster. Proc Natl Acad Sci U S A. 2013;110(42):17065-70. Epub 2013/10/02. doi: 1313258110 [pii]

10.1073/pnas.1313258110. PubMed PMID: 24082142; PubMed Central PMCID: PMC3801025.

10. Leipold F, Wardenga R, Bornscheuer UT. Cloning, expression and characterization of a eukaryotic cycloalkanone monooxygenase from Cylindrocarpon radicicola ATCC 11011. Appl Microbiol Biotechnol. 2012;94(3):705-17. doi: 10.1007/s00253-011-3670-z. PubMed PMID: 22075635.

11. Leisch H, Shi R, Grosse S, Morley K, Bergeron H, Cygler M, et al. Cloning, Baeyer-Villiger biooxidations, and structures of the camphor pathway 2-oxo-Delta(3)-4,5,5-trimethylcyclopentenylacetyl-coenzyme A monooxygenase of Pseudomonas putida ATCC 17453. Appl Environ Microbiol. 2012;78(7):2200-12. Epub 2012/01/24. doi: AEM.07694-11 [pii]

10.1128/AEM.07694-11. PubMed PMID: 22267661; PubMed Central PMCID: PMC3302634.

12. Kostichka K, Thomas SM, Gibson KJ, Nagarajan V, Cheng Q. Cloning and characterization of a gene cluster for cyclododecanone oxidation in Rhodococcus ruber SC1. J Bacteriol. 2001;183(21):6478-86. doi: 10.1128/JB.183.21.6478-6486.2001. PubMed PMID: 11591693; PubMed Central PMCID: PMC100144.

13. Iwaki H, Wang S, Grosse S, Bergeron H, Nagahashi A, Lertvorachon J, et al. Pseudomonad cyclopentadecanone monooxygenase displaying an uncommon spectrum of Baeyer-Villiger oxidations of cyclic ketones. Appl Environ Microbiol. 2006;72(4):2707-20. doi: 10.1128/AEM.72.4.2707-2720.2006. PubMed PMID: 16597975; PubMed Central PMCID: PMC1449013.

14. Kamerbeek NM, Moonen MJ, Van Der Ven JG, Van Berkel WJ, Fraaije MW, Janssen DB. 4-Hydroxyacetophenone monooxygenase from Pseudomonas fluorescens ACB. A novel flavoprotein catalyzing Baeyer-Villiger oxidation of aromatic compounds. Eur J Biochem. 2001;268(9):2547-57. PubMed PMID: 11322873.

15. Wen Y, Hatabayashi H, Arai H, Kitamoto HK, Yabe K. Function of the cypX and moxY genes in aflatoxin biosynthesis in Aspergillus parasiticus. Appl Environ Microbiol. 2005;71(6):3192-8. doi: 10.1128/AEM.71.6.3192-3198.2005. PubMed PMID: 15933021; PubMed Central PMCID: PMC1151844.
