# Supplementary material for: The Origin and Evolution of Baeyer—Villiger Monooxygenases (BVMOs): An Ancestral Family of Flavin Monooxygenases
Source: PLoS One. 2015 Jul 10;10(7):e0132689. doi: 10.1371/journal.pone.0132689 (PMC4498894; doi:10.1371/journal.pone.0132689)
Supplement: S4 File — (PDF) [file pone.0132689.s014.pdf]

Conserved domain architecture analysis of identified BVMO sequences

A preliminary analysis was conducted in order to determine if BVMOs cluster together on the basis of their conserved domain architecture. The NCBI tool CDART (Conserved Domain Architecture Retrieval Tool, available at: http://www.ncbi.nlm.nih.gov/Structure/lexington/lexington.cgi) was employed [1, 2]. Full BVMO encoding sequences were submitted.

All sequences share a Rossmann-fold NAD(P)H/NAD(P)($^+$) binding (NADB) domain (cl21454) as expected. This domain is found in numerous redox enzymes and it exhibits a consensus binding pattern similar to GXGXXG. Typically enzymes belonging to this superfamily display two NADB domains. Among all analyzed BVMOs only two sequences showed extra domains. BVMO$_{Af1}$ (XP_747160) from *A. fumigatus* displays a C-terminal methyltransferase specific domain (cl17173: AdoMet_MTases) which is predicted to be part of a PKS enzyme complex. Likewise, sequence Ehux5 (XP_005761505) from *E. huxleyi* displays an N-terminal RhaT multidomain (COG0697) predicted to be a permease involved in the drug/metabolite transporter (DMT) superfamily. Due to the location of these two BVMOs in the phylogenetic tree (Fig. 2), it seems likely possible that the fusion event of the NADB domain to the other catalytic domains was lineage-specific.

Based on these results, it is not possible to state that BVMOs cluster in the phylogenetic tree according to their different domain architectures, since all display the same basic architecture. However, the use of more sensitive methodologies including structural data might provide further insight in the domain architecture analysis of these sequences.

1. Geer LY, Domrachev M, Lipman DJ, Bryant SH. CDART: protein homology by domain architecture. Genome Res. 2002;12(10):1619-23. doi: 10.1101/gr.278202. PubMed PMID: 12368255; PubMed Central PMCID: PMC187533.

2. Marchler-Bauer A, Derbyshire MK, Gonzales NR, Lu S, Chitsaz F, Geer LY, et al. CDD: NCBI's conserved domain database. Nucleic Acids Res. 2015;43(Database issue):D222-6. doi: 10.1093/nar/gku1221. PubMed PMID: 25414356; PubMed Central PMCID: PMC4383992.
