# Supplementary material for: The Origin and Evolution of Baeyer—Villiger Monooxygenases (BVMOs): An Ancestral Family of Flavin Monooxygenases
Source: PLoS One. 2015 Jul 10;10(7):e0132689. doi: 10.1371/journal.pone.0132689 (PMC4498894; doi:10.1371/journal.pone.0132689)
Supplement: S1 Table — All BVMOs protein sequences employed in this work are depicted. Colors are used to display the origin of each sequence, as follows: Bacteria (white), Fungi (grey), Green plants and Rhodophyta (green), Haptophyta (light purple), Metazoa (orange) and Archaea (blue). Sequences marked as (*) have been previously characterized. Sequences marked as (#) have been previously named as AFL838, AFL619, AFL210 and AFL456, respectively [20]. Sequences without marks are reported here for the first time as putative BVMOs. Most of these sequences are derived from automated annotation of genomic data. (PDF) [file pone.0132689.s015.pdf]

| Name | Protein Id. | Organism |
|---|---|---|
| HAPMO* | AAK54073.1 | *Pseudomonas fluorescens* |
| HAPMO* | ACJ37423.1 | *Pseudomonas putida* |
| CHMO* | AAG10021.1 | *Acinetobacter* sp. SE19 |
| CHMO* | AAG01289.1 | *Brevibacterium* sp. HCU |
| CHMO* | BAH56677.1 | *Rhodococcus* sp. HI-31 |
| CHMO* | ABQ10653.1 | *Arthrobacter* sp. L661 |
| CPDMO* | BAN84077 | *Pseudomonas* sp. HI-70 |
| PAMO* | YP_289549 | *Thermobifida fusca* |
| STMO* | BAA24454 | *Rhodococcus rhodochrous* |
| EthA* | NP_218371.1 | *Mycobacterium tuberculosis* H37Rv |
| CPMO* | BAC22652.1 | *Comamonas* sp. NCIMB 9872 |
| CDMO* | AAL14233.1 | *Rhodococcus ruber* |
| MEKMO* | ABI15711 | *Pseudomonas veronii* |
| OTEMO* | H3JQW0.1 | *Pseudomonas putida* |
| ACMO* | BAF43791.1 | *Gordonia* sp. TY-5 |
| CAMO* | AET80001.1 | *Cylindrocarpon radicicola* |
| BVMOAf1* | XP_747160.1 | *Aspergillus fumigatus* Af293 |
| BVMOAf2 | XP_746949.1 | *Aspergillus fumigatus* Af293 |
| BVMOAf3 | XP_755274.1 | *Aspergillus fumigatus* Af293 |
| CmBVMO* | BAM80902.1 | *Cyanidioschyzon merolae* strain 10D |
| PpBVMO* | XP_001758613.1 | *Physcomitrella patens* |
| BVMOAPar | AAS66023 | *Aspergillus parasiticus* |
| Afum1 | XP_756084 | *Aspergillus fumigatus* |
| Afum2 | XP_752204 | *Aspergillus fumigatus* |
| Afum3 | XP_751255 | *Aspergillus fumigatus* |
| Afum4 | XP_747774 | *Aspergillus fumigatus* |
| Afum5 | XP_754119 | *Aspergillus fumigatus* |
| Afla1 | XP_002382393 | *Aspergillus flavus* |
| Afla2 | XP_002379630 | *Aspergillus flavus* |
| Afla3 | XP_002377628 | *Aspergillus flavus* |
| Afla4 | XP_002378661 | *Aspergillus flavus* |
| Afla5 | XP_002374045 | *Aspergillus flavus* |
| Afla6 | AAS90037 | *Aspergillus flavus* |
| Afla7 | AAS90108 | *Aspergillus flavus* |
| Afla8 | AAS90083 | *Aspergillus flavus* |
| Afla9 | AAS90015 | *Aspergillus flavus* |
| Afla10 | XP_002379905 | *Aspergillus flavus* |
| Afla11 | XP_002382061 | *Aspergillus flavus* |

| | | |
|---|---|---|
| Afla12 | XP_002372173 | *Aspergillus flavus* |
| Afla13 | XP_002379194 | *Aspergillus flavus* |
| Afla14 | XP_002378632 | *Aspergillus flavus* |
| Afla15 | XP_002381778 | *Aspergillus flavus* |
| Afla16 | XP_002378022 | *Aspergillus flavus* |
| Afla17 | XP_002377626 | *Aspergillus flavus* |
| Afla18 | XP_002379903 | *Aspergillus flavus* |
| Afla19 | XP_002377590 | *Aspergillus flavus* |
| Afla20*# | XP_002375657 | *Aspergillus flavus* |
| Afla21*# | XP_002383043 | *Aspergillus flavus* |
| Afla22*# | XP_002375343 | *Aspergillus flavus* |
| Afla23*# | XP_002375466 | *Aspergillus flavus* |
| Afla24 | XP_002384949 | *Aspergillus flavus* |
| Afla25 | XP_002381394 | *Aspergillus flavus* |
| Afla26 | XP_002378456 | *Aspergillus flavus* |
| Afla27 | XP_002382416 | *Aspergillus flavus* |
| Afla28 | XP_002380575 | *Aspergillus flavus* |
| Anig1 | XP_001400968.1 | *Aspergillus niger* |
| Anig2 | XP_001394629.2 | *Aspergillus niger* |
| Anig3 | XP_001396319.2 | *Aspergillus niger* |
| Anig4 | XP_001390523.2 | *Aspergillus niger* |
| Anig5 | XP_001394771.1 | *Aspergillus niger* |
| Anig6 | XP_001395789.2 | *Aspergillus niger* |
| Anig7 | XP_001395571.1 | *Aspergillus niger* |
| Anig9 | XP_001400792.1 | *Aspergillus niger* |
| Ylip1 | XP_502954.1 | *Yarrowia lipolytica* |
| Ylip2 | XP_503686.1 | *Yarrowia lipolytica* |
| Ylip3 | XP_503445.1 | *Yarrowia lipolytica* |
| Ylip4 | XP_505928.1 | *Yarrowia lipolytica* |
| Ylip5 | Q6C083 | *Yarrowia lipolytica* |
| Ylip6 | XP_503818.1 | *Yarrowia lipolytica* |
| Umay1 | XP_757861.1 | *Ustilago maydis* |
| Umay2 | XP_756855.1 | *Ustilago maydis* |
| Umay3 | XP_759562.1 | *Ustilago maydis* |
| Umay4 | XP_761959.1 | *Ustilago maydis* |
| BVMO | XP_007845070.1 | *Moniliophthora roreri* |
| BVMO | XP_007388658.1 | *Punctularia strigosozonata* |
| BVMO | XP_007771996.1 | *Coniophora puteana* |
| BVMO | XP_007348198.1 | *Auricularia delicata* |
| BVMO | XP_007860503.1 | *Gloeophyllum trabeum* |

| | | |
|---|---|---|
| Phod1 | XP_005978382 | *Pantholops hodgsonii* |
| Phod2 | XP_005978142 | *Pantholops hodgsonii* |
| Phod3 | XP_005978883 | *Pantholops hodgsonii* |
| Phod4 | XP_005973192 | *Pantholops hodgsonii* |
| Odio1 | CBY14169.1 | *Oikopleura dioica* |
| Odio3 | CBY40950.1 | *Oikopleura dioica* |
| BVMO | XP_002164118.2 | *Hydra vulgaris* |
| TrkA | ACD54725.1 | *Adineta vaga* |
| Ehux1 | XP_005784821 | *Emiliania huxleyi* |
| Ehux2 | XP_005760720 | *Emiliania huxleyi* |
| Ehux3 | XP_005788719 | *Emiliania huxleyi* |
| Ehux4 | XP_005769131 | *Emiliania huxleyi* |
| Ehux5 | XP_005761505 | *Emiliania huxleyi* |
| BVMO | BAK01240.1 | *Hordeum vulgare* |
| BVMO | WP_012944942.1 | *Haloterrigena turmenika* |
| BVMO | WP_007260850.1 | *Natronolimnobius innermongolicus* |
| BVMO | WP_021051163.1 | *Haloquadratum walsbyi* |
| CHMO | WP_024517297.1 | *Bradyrhizobium* sp. Tv2a-2 |
| CHMO | WP_011502279.1 | *Rhodopseudomonas palustris* |
| CHMO | WP_020737909.1 | *Sorangium cellulosum* |
| CHMO | ETW99872.1 | *Candidatus Entotheonella* sp. TSY1 |
| BVMO | YP_723341.1 | *Trichodesmium erythraeum* IMS101 |
| BVMO | WP_008190503.1 | *Moorea producens* |
| BVMO | WP_019504934.1 | *Pleurocapsa* sp. PCC 7319 |
| BVMO | WP_019009166.1 | *Deinococcus aquatilis* |
| BVMO | WP_019587858.1 | *Deinococcus apachensis* |
| BVMO | EYB69487.1 | *Deinococcus phoenicis* |
| HAPMO | WP_007915698.1 | *Ktedonobacter racemifer* |
| BVMO | WP_012121543.1 | *Roseiflexus castenholzii* |
| BVMO | WP_011957134.1 | *Roseiflexus* sp. RS-1 |
| HAPMO | YP_007323166.1 | *Fibrella aestuarina* BUZ 2 |
| Srub1 | YP_003570194.1 | *Salinibacter ruber* M8 |
| Srub2 | YP_444391.1 | *Salinibacter ruber* DSM 13855 |
| BVMO | WP_017730895.1 | *Nafulsella turpanensis* |
| BVMO | WP_016546615.1 | *Leptospira wolffii* |
| HAPMO | WP_008589837.1 | *Leptospira licerasiae* |
| HAPMO | WP_014803102.1 | *Turneriella parva* |
| HAPMO | YP_004267987.1 | *Planctomyces brasiliensis* DSM 5305 |
| BVMO | EWG10346.1 | *Bacillus firmus* DS1 |
| BVMO | WP_018708010.1 | *Bacillus fordii* |

| | | |
|---|---|---|
| BVMO | WP_021289645.1 | *Virgibacillus* sp. CM-4 |
| BVMO | CAN95107.1 | *Sorangium cellulosum* So ce56 |
| CHMO | ETR78797.1 | *Afipia* sp. P52-10 |
