# Supplementary material for: The Origin and Evolution of Baeyer—Villiger Monooxygenases (BVMOs): An Ancestral Family of Flavin Monooxygenases
Source: PLoS One. 2015 Jul 10;10(7):e0132689. doi: 10.1371/journal.pone.0132689 (PMC4498894; doi:10.1371/journal.pone.0132689)
Supplement: S2 Table — (PDF) [file pone.0132689.s016.pdf]

Name	Protein Id.	Organism	Type	Class
FMO2	XP_005957292.1	*Pantholops hodgsonii*	FMO	B
FMO6	XP_005973473.1	*P. hodgsonii*	FMO	B
FMO3	XP_005973472.1	*P. hodgsonii*	FMO	B
FMO4	XP_005957289.1	*P. hodgsonii*	FMO	B
FMO5	XP_005960160.1	*P. hodgsonii*	FMO	B
FMO2	XP_002164616.1	*Hydra vulgaris*	FMO	B
FMO4	XP_004207563.1	*Hydra vulgaris*	FMO	B
FMO1	XP_001776377.1	*Physcomitrella patents*	FMO	B
FMO2	XP_001753831.1	*P. patents*	FMO	B
FMO1	NP_001052088.1	*Oryza sativa*	FMO	B
FMO2	NP_001066335.2	*O. sativa*	FMO	B
FMO	XP_758300.1	*Ustilago maydis*	FMO	B
FMO2-2	NP_001068630.1	*Bos taurus*	FMO	B
FMO6-X1	XP_002694062.1	*B. taurus*	FMO	B
FMO4	XP_004013740.1	*Ovis aries*	FMO	B
FMO3	XP_003130142.2	*Sus crofa*	FMO	B
FMO4	XP_004599240.1	*Ochotona princeps*	FMO	B
FMO5	XP_004589355.1	*O. princeps*	FMO	B
FMO3	NP_008825.4	*Homo sapiens*	FMO	B
FMO2	NP_001451.1	*H. sapiens*	FMO	B
FMORabliv	AAB19844.1	*Rabbit*	FMO	B
FMO5	NP_001075714.1	*Oryctolagus cuniculus*	FMO	B
FMO1	NP_036924.1	*Rattus norvegicus*	FMO	B
FMOGS-OX1	NP_176761.1	*Arabidopsis thaliana*	FMO	B
FMOGS-OX7	NP_176446.1	*A. thaliana*	FMO	B
FMOA	YP_700733.1	*Rhodococcus jostii*	FMO	B
FMOB	YP_703295.1	*R. jostii*	FMO	B
FMOG	YP_705633.1	*R. jostii*	FMO	B
FMOH	YP_707856.1	*R. jostii*	FMO	B
FMO5	EKC23885.1	*Crassostrea gigas*	FMO	B
FMO2	XP_002934193.2	*Xenopus laevis*	FMO	B
FMO5	XP_004651790.1	*Jaculus jaucus*	FMO	B
FMO5	XP_005370168.1	*Microtus ochrogaster*	FMO	B
FMO2-3	XP_007088787.1	*Panthera tigris*	FMO	B
YUCCA	JAC76550.1	*Tetraselmis* sp. GSL018	FMO	B
YUCCA	AAL23750.1	*Arabidopsis thaliana*	FMO	B
YUCCA	XP_002515593.1	*Ricinus communis*	FMO	B
YUCCA	XP_007013782.1	*Theobroma cacao*	FMO	B

YUCCA4	XP_004138744.1	*Cucumis sativus*	FMO	B
YUCCA2	AFG16915.1	*Fragaria ananassa*	FMO	B
FMO	WP_027026289.1	*Mesorhizobium* sp. URHA0056	FMO	B
FMO	WP_031357647.1	*Burkholderia sordidicola*	FMO	B
FMO	WP_018619989.1	*Spirosoma luteum*	FMO	B
FMO	WP_015330005.1	*Fibrella aestuarina*	FMO	B
FMO	WP_007825618.1	*Streptomyces* sp. Tu6071	FMO	B
FMO	CCQ36834.1	*Natronomonas moolapensis* 8.8.11	FMO	B
NMO	YP_704660.1	*R. jostii*	NMO	B
NMO	NP_251076.1	*Pseudomonas aeruginosa*	NMO	B
LucD	YP_444061.1	*Escherichia coli*	NMO	B
NMOSidA	XP_755103.1	*Aspergillus fumigatus*	NMO	B
NMORhbE	AAK65920.1	*Sinorhizobium meliloti*	NMO	B
ALCA	Q44740.2	*Bordetella bronchiseptica*	NMO	B
SID1	P56584.2	*Ustilago maydis*	NMO	B
NMO	XP_007456134.1	*Lipotes vexillifer*	NMO	B
NMO	XP_005982544.1	*P. hodgsonii*	NMO	B
NMO	XP_005964211.1	*Mesorhizobium sp*	NMO	B
NMO	AGB33522.1	*Natrinema pellirubrum* DSM 15624	NMO	B
NMO	CAP15481.1	*Halobacterium salinarum* R1	NMO	B
4HB3MO	YP_001238756.1	*Bradyrhizobium* sp. BTAi1	Hydroxylase	A
2,4DCP6MO	AGI86992.1	*Streptomyces albus* J1074	Hydroxylase	A
HPXO	B5B0J6.1	*Klebsiella oxytoca*	Hydroxylase	A
RiMO	AAB41059.1	*Rhodococcus hoagii*	Hydroxylase	A
