# Supplementary material for: The Origin and Evolution of Baeyer—Villiger Monooxygenases (BVMOs): An Ancestral Family of Flavin Monooxygenases
Source: PLoS One. 2015 Jul 10;10(7):e0132689. doi: 10.1371/journal.pone.0132689 (PMC4498894; doi:10.1371/journal.pone.0132689)
Supplement: S3 Table — (PDF) [file pone.0132689.s017.pdf]

Seq. name	Organism	Primer sequences	Amplicon size	Ta°C
Afla10	*Aspergillus flavus*	Fw_int_*Afla10*: GTAYGGYGACGAARTCCACAA<br>Rev_int_*Afla10*: TCCACCAAACARCGCKGTGA	103 bp	51
BVMO	*Hordeum vulgare*			
β-tubulin	*Aspergillus flavus*	Fw_*TUBB*: GGTAACCAAATAGGTGCCGCT<br>Rev_*TUBB*: CATCCAGAGCAAGAACCAGACCTA	1300 bp	62
α-tubulin	*Hordeum vulgare*	Fw_*TUBA*: TTCGCCCGTGGTCATTACA<br>Rev_*TUBA*: GCATTGAAGACAAGGAAGCCC	113 bp	54
Ehux1	*Emiliania huxleyi*	Fw_int_*Ehux1-2*: CCCAGCAAAGTCCTTTCTTCT<br>Rev_int_*Ehux1-2*: CGATGACAACCACCCTCTTC	588 bp	51
Ehux2	*Emiliania huxleyi*			
Ehux3	*Emiliania huxleyi*	Fw_int_*Ehux3-4*: GCCGAACCTCTTCTACATCTT<br>Rev_int_*Ehux3-4*: CAGAGTCAGAGAAAGGACGATAC	441 bp	51
Ehux4	*Emiliania huxleyi*			
Ehux5	*Emiliania huxleyi*	Fw_int_*Ehux5*: TGCCTCGACACAGACTACTA<br>Rev_int_*Ehux5*: CACGATACCTGCGATGATGT	437 bp	51

To amplify both *Afla10* (from *A. flavus*) and *BVMO* (from *H. vulgare*) sequences, degenerated primers were designed. As control, primers to amplify the housekeeping genes *β-tubulin* [1] and *α-tubulin* [2] were used.

To study the presence of BVMOs sequences in *E. huxleyi*, primers were designed to amplify the conserved region of *Ehux1* and *Ehux2* genes, as well as *Ehux3* and *Ehux4*. For *Ehux5* a specific pair of primers was designed.

1. Sweeney MJ, Pamies P, Dobson AD. The use of reverse transcription-polymerase chain reaction (RT-PCR) for monitoring aflatoxin production in *Aspergillus parasiticus* 439. Int J Food Microbiol. 2000;56(1):97-103. PubMed PMID: 10857929.
2. Jarosova J, Kundu JK. Validation of reference genes as internal control for studying viral infections in cereals by quantitative real-time RT-PCR. BMC Plant Biol. 2010;10:146. doi: 10.1186/1471-2229-10-146. PubMed PMID: 20630112; PubMed Central PMCID: PMC3095291.
